# Mechanisms of Left-Right Coordination in Mammalian Locomotor Pattern Generation Circuits: A Mathematical Modeling View

**DOI:** 10.1371/journal.pcbi.1004270

**Published:** 2015-05-13

**Authors:** Yaroslav I. Molkov, Bartholomew J. Bacak, Adolfo E. Talpalar, Ilya A. Rybak

**Affiliations:** 1 Department of Mathematical Sciences, Indiana University—Purdue University, Indianapolis, Indiana, United States of America; 2 Department of Neurobiology and Anatomy, Drexel University College of Medicine, Philadelphia, Pennsylvania, United States of America; 3 Department of Neuroscience, Karolinska Institute, Stockholm, Sweden; Northeastern University, UNITED STATES

## Abstract

The locomotor gait in limbed animals is defined by the left-right leg coordination and locomotor speed. Coordination between left and right neural activities in the spinal cord controlling left and right legs is provided by commissural interneurons (CINs). Several CIN types have been genetically identified, including the excitatory V3 and excitatory and inhibitory V0 types. Recent studies demonstrated that genetic elimination of all V0 CINs caused switching from a normal left-right alternating activity to a left-right synchronized “hopping” pattern. Furthermore, ablation of only the inhibitory V0 CINs (V0_D_ subtype) resulted in a lack of left-right alternation at low locomotor frequencies and retaining this alternation at high frequencies, whereas selective ablation of the excitatory V0 neurons (V0_V_ subtype) maintained the left–right alternation at low frequencies and switched to a hopping pattern at high frequencies. To analyze these findings, we developed a simplified mathematical model of neural circuits consisting of four pacemaker neurons representing left and right, flexor and extensor rhythm-generating centers interacting via commissural pathways representing V3, V0_D_, and V0_V_ CINs. The locomotor frequency was controlled by a parameter defining the excitation of neurons and commissural pathways mimicking the effects of N-methyl-D-aspartate on locomotor frequency in isolated rodent spinal cord preparations. The model demonstrated a typical left-right alternating pattern under control conditions, switching to a hopping activity at any frequency after removing both V0 connections, a synchronized pattern at low frequencies with alternation at high frequencies after removing only V0_D_ connections, and an alternating pattern at low frequencies with hopping at high frequencies after removing only V0_V_ connections. We used bifurcation theory and fast-slow decomposition methods to analyze network behavior in the above regimes and transitions between them. The model reproduced, and suggested explanation for, a series of experimental phenomena and generated predictions available for experimental testing.

## Author Summary

Movements of left and right limbs in mammals during locomotion are controlled by distinct rhythm-generating neuronal circuits in the spinal cord. Complex interactions between these circuits provide flexible coordination of limb movements in different gaits. It was shown that interactions between left and right spinal circuits are mediated by commissural interneurons. Genetic ablation of a particular type of these interneurons, called V0, leads to switching from a regular, left-right alternating “walking” activity to a left-right synchronous “hopping” pattern. Moreover, the V0 commissural interneurons have excitatory and inhibitory subtypes that appear to play different roles in the left-right coordination depending on locomotor speed. In this theoretical study, we build a simplified mathematical model of spinal circuits that describes left and right rhythm generators interacting bilaterally via several types of commissural connections. Using this model, we simulate different experimental manipulations, analyze the resultant alternating and synchronous regimes of activity, and propose explanations for the results of experimental studies. We show that although both excitatory and inhibitory V0 commissural pathways support left-right alternation, the resultant locomotor pattern and gait depend on the balance between different commissural interactions, which in turn may depend on the level of neuronal excitation and locomotor speed.

## Introduction

Central Pattern Generators (CPGs) are neural networks that can produce organized rhythmic motor activities in the absence of rhythmic inputs and feedbacks from other parts of the nervous system. The CPGs that generate basic rhythmic locomotor activities and control locomotion in vertebrates are located in the spinal cord [1–11]. It appears that each limb in mammals is controlled by a separate CPG because cats with chronic thoracic spinal lesions were shown to step on a split treadmill with different speed for the left and right hind limbs [12]. The gait of locomotion is defined by the coordinated limb movements and hence by the phase relationships between rhythmic patterns generated by CPGs controlling each limb. In turn, these relationships are defined by neural circuits within the spinal cord providing direct or indirect interactions between the CPGs.

Several computational models of neural circuits were proposed to reproduce and explain the left-right and segmental coordination during locomotion in lamprey [13–16] and left-right hindlimb and quadrupedal limb coordination in the salamander [17–19] and mammals [20–22]. Some computational studies focused on gait transitions analyzing them as bifurcation phenomena [23–25]. Spinal circuits in some models were simulated and analyzed as coupled nonlinear oscillators [26–28]. In these models, each oscillator represented an independent rhythm-generating center (simulated as a single neuron or a neural population), and the main goal of these models was to study how inter-oscillator couplings could affect the synchronization and the phase lags between rhythm-generating centers [18]. It is considered that the locomotor gait and pattern in these models mostly depend on the topology of interactions and properties of neurons involved, rather than on the local rhythm-generation mechanisms [18]. However, even in lower vertebrates such as the lamprey, it is extremely difficult to identify and characterize all neurons and neural circuits that are involved in such coupling/interactions during locomotion.

It is generally considered that phase relationships between the activities of corresponding neurons on the left and right sides of the spinal cord are provided by so-called commissural interneurons (CINs), whose axons cross the midline and innervate neurons on the contralateral side of the cord [6, 29–32]. There are different types of inhibitory and excitatory CINs, which are involved in the left-right alternating (e.g., during normal walking) or left-right synchronized (e.g., during hopping or galloping) locomotor activities [6–8,29–32].

Significant progress in understanding the functional and structural organization of spinal circuits has been achieved due to recently developed combinations of genetic, molecular, and developmental techniques. Several populations of neurons were found to be derived from genetically distinct populations of embryonic neurons in the spinal cord of adult mice [33–36]. Some of these genetically characterized neurons were identified as CINs, including the V0 and V3 interneurons [6,30,33,37,38]. The V0 population contains both excitatory and inhibitory CINs that both contribute to alternating left and right neuronal activities [6,30,37]. The V3 neurons are excitatory and mediate interactions promoting left-right synchronized hopping behavior [38].

The V0 population contains genetically distinct subpopulations of CINs: V0_V_ and V0_D_. The inhibitory V0_D_ neurons constitute about two-thirds of the V0 population and the excitatory V0_V_ neurons constitute about one-third [6,30,39]. The specific functional roles of V0_V_ and V0_D_ subpopulations in coordinating left and right locomotor activities have been recently studied using isolated spinal cord preparations from genetically transformed mice with either V0_V_, or V0_D_, or both V0 subpopulations knocked out [37]. The spinal cord was isolated *in vitro* to ensure that supraspinal structures were not participating in rhythm generation. The specific contribution of each subpopulation to the left-right coordination has been found to depend on the frequency of locomotor oscillations. Locomotor oscillations were induced by application of a mixture of N-methyl-D-aspartate (NMDA) and 5-hydroxytryptamine (5-HT). These oscillations were characterized by alternating activities recorded from left and right flexor-related (LL2 and RL2) and left and right extensor-related (LL5 and RL5) lumbar roots. The speed of locomotion (oscillation frequency) was regulated by the NMDA concentration [37,40]. Measuring the phase differences between rhythmic activities recorded from LL2 and RL2 roots and from LL5 and RL5 roots allowed for identification of an alternating or synchronized pattern. The results of these studies can be summarized as follows [37]: (i) spinal cord preparations from control mice showed left-right alternating behavior at all locomotor frequencies; (ii) preparations from the mice with genetically ablated V0 populations (both V0_V_ and V0_D_ types) showed synchronized (hopping) activity at all locomotor frequencies; (iii) preparations from the mice with only V0_V_ populations ablated maintained left-right alternation at low frequencies but switched to hopping gait at high frequencies; (iv) preparations from the mice with selectively ablated V0_D_ populations showed hopping at low frequencies and switched to alternation at high frequencies.

This model represents a simplified version of the large-scale model [41], which was used for computer simulation of spinal circuits involved in rhythm generation and frequency-dependent left-right CIN-mediated interactions, but was too complicated to use methods of qualitative theory of differential equations and dynamical systems. Therefore, in this study, we formulated and analyzed a simplified mathematical model. The model consisted of four pacemaker neurons representing left and right, flexor and extensor rhythm-generating centers interacting via commissural pathways representing V3, V0_D_, and V0_V_ CINs. We used this model to investigate different regimes of behavior of the intact circuitry, and following a removal of commissural interactions mediated by V0_D_, or V0_V_, or both V0 CIN pathways to mimic recent experimental studies of Talpalar et al. [37]. Then, using methods of bifurcation theory and fast-slow decomposition, we analyzed the essential properties of this system under different conditions and propose plausible explanations for the experimentally observed behavioral transformations.

## Methods

### Model Formulation

Most of the recent models of mammalian spinal circuits and locomotor CPGs were based on interacting populations of tens or hundreds of spiking neurons modeled in the Hodgkin-Huxley style and described by 5–10 differential equations per neuron [41–46]. Such large networks of spiking neurons are too complex to be analyzed by traditional mathematical approaches, and hence certain simplifications need to be applied. Such a simplification has been previously proposed in the analysis of the model of the mammalian respiratory CPG [47,48]. In these studies, each population of spiking neurons was described by a single, non-spiking neuron model, in which the voltage variable represented an average voltage for the population and the output activity was described as a nonlinear function of voltage, *f*(*V*) [47–49]. It was established that such a simplification can provide a reasonably accurate description of population activity, including transitions between quiescent (silent) and active states in population dynamics [47]. This simplification was used in our present model.

The other simplification of the current study was that, in contrast to the previous multi-scale models [42–45], we focused only on rhythm-generating circuits and did not consider motoneurons and interneurons (such as Ia and Renshaw cells) that are not critically involved in rhythmogenesis and left-right coordination. Therefore, the reduced neural circuit that we analyzed included two (left and right) rhythm generators (RGs) bilaterally interconnected via several commissural pathways.

**Fig 1:**
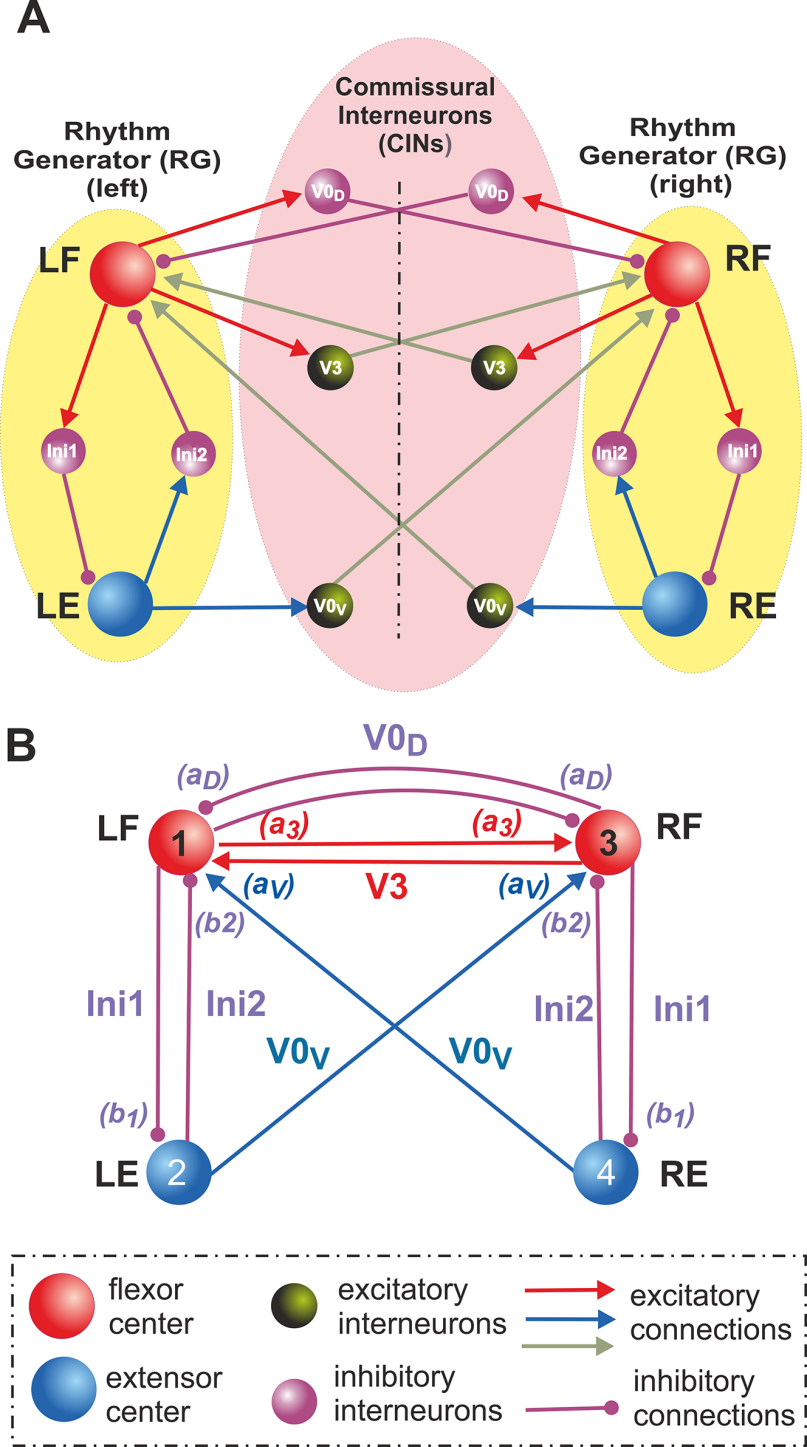
Organization of locomotor CPGs and commissural interactions. **A**. Hypothetical schematic of flexor-extensor and commissural left-right interactions between the locomotor centers. Rhythm generator (RG) in each side of the cord consists of one flexor (left, LF, and right, RF) and one extensor (left, LE, and right, RE) center that reciprocally inhibit each other via inhibitory populations of neurons (Ini1 and Ini2 on each side). Left and right RGs interact via populations of commissural interneurons (CINs). In the proposed model, three types of CINs are considered: excitatory V3 that mediate excitatory connections between LF and RF, inhibitory V0_D_ that mediate inhibitory connections between LF and RF, and excitatory V0_V_, that mediate excitatory inputs from each extensor center (LE and RE) to the contralateral flexor center (RF and LF, respectively). **B.** The schematic of simplified model, in which all interneuron populations, including CINs, are replaced by the corresponding pathways with particular synaptic weights. See text for details.

Each RG consisted of one flexor (left, LF, or right, RF) and one extensor (left, LE, or right, RE) centers reciprocally inhibiting each other via inhibitory interneurons (Ini1 and Ini2, respectively) (Fig 1A). Three major commissural pathways were considered: (1) the excitatory V3 CINs mediate mutual excitation between the left and right flexor centers (LF and RF); (2) the inhibitory V0_D_ CINs mediate mutual inhibition between these centers; and (3) the excitatory V0_V_ CINs that were also shown to contribute to mutual inhibition between these centers and promote left-right alternation.

To provide mutual inhibition, the excitatory V0_V_ CINs should either receive inputs from the ipsilateral flexor centers and affect the contralateral flexor centers via inhibitory interneurons (as suggested in [37,41]) or mediate a crisscross excitation from each extensor center to the contralateral flexor center as shown in Fig 1A (see also [41]). Because no recordings from V0_V_ neurons have been made so far, it is difficult to determine which of the two V0_V_ pathways suggested is more realistic or whether both these pathways are present in the spinal cord (see discussion in [41]). Preliminary large-scale simulations have shown that both pathways lead to qualitatively similar behavior [41]. Therefore, in this study, we only focused on the V0_V_ CIN pathways providing the direct crisscross excitation from each extensor center to the contralateral flexor center (Fig 1A).

The final simplification concerned all interneurons (i.e. CINs, Ini1, and Ini2), which, for simplicity, were removed and replaced by the corresponding direct synaptic connections as shown in Fig 1B. Therefore, the final simplified model under investigation consisted of four centers (LF, LE, RF, and RE) with the unilateral inhibitory interactions between flexor and extensor centers on each side via Ini1 and Ini2 pathways and the bilateral V3, V0_D_, and V0_V_ commissural interactions between the left and right centers as shown in Fig 1B.

### Rhythm-Generating Centers and Connections

Each center is described using a reduced activity-based non-spiking neuron model with only two dynamical variables. This model was adapted from a reduced model of the population of intrinsically bursting neurons in the pre-Bötzinger complex developed by Rubin et al. [47,48]. Endogenous bursting in these neurons was suggested to involve the persistent (slowly inactivating) sodium current, *I*_*NaP*_, first described by Butera et al. [50] (Model 1), with burst termination based on the slow inactivation of this current. This model was shown able to generate busting activity with a wide range of a parameters defining neuronal excitation and burst frequencies, and exhibited the correct change (reduction) of burst amplitude when the frequency increased [47,48]. This model has been successfully implemented in several previous models of the locomotor CPG [41–46,51,52]. The persistent sodium current was indeed found in spinal interneurons [52–56] and its blockade terminated rhythm generation in the rat spinal cord [53].

Similar to the Rubin et al. models [47,48], our formulation for each center includes an explicit representation of *I*_*NaP*_:

$$C\cdot \dot{V}=-I_{NaP}-I_{L},$$ (1)

where *C* is the membrane capacitance, *I*_*NaP*_ represents the persistent sodium current, and *I*_*L*_ is the leak current. The currents in Eq (1) are described as follows:

$$I_{NaP}={\bar{g}}_{NaP}\cdot m_{NaP\infty }(V)\cdot h_{NaP}\cdot (V-E_{Na});$$ (2)

$$I_{L}={\bar{g}}_{L}\cdot (V-E_{L}),$$ (3)

where ${\bar{g}}_{NaP}$ and ${\bar{g}}_{L}$ are the maximal conductances of the persistent sodium and leak channels, and *E*_*Na*_ and *E*_*L*_ are the reversal potentials for sodium and leak currents, respectively. *h*_*NaP*_ is the *I*_*NaP*_ inactivation gating variable, and *m*_*NaP∞*_ (*V*) represents voltage-dependent steady state of *I*_*NaP*_ activation:

$$m_{NaP\infty }(V)={\left(1+\text{exp}\left\{(V-V_{mNaP})/{\text{k}}_{mNaP}\right\}\right)}^{-1};$$ (4)

The *I*_*NaP*_ activation, *m*_*NaP∞*_ (*V*), is considered instantaneous; the *I*_*NaP*_ inactivation is slow and *h*_*NaP*_ represents the “slow” dynamical variable in this model described as follows:

$$\tau_{hNaP}(V)\cdot {\dot{h}}_{NaP}=h_{NaP\infty }(V)-h_{NaP},$$ (5)

where *h*_*NaP∞*_ (*V*) and *τ*_*hNaP*_ (*V*) represent the voltage-dependent steady state and time constant for inactivation, respectively:

$$h_{NaP\infty }(V)={\left(1+\text{exp}\left\{(V-V_{hNaP})/{\text{k}}_{hNaP}\right\}\right)}^{-1}.$$ (6)

$$\tau_{hNaP\infty }(V)=\tau_{hNaP\text{max}}/\text{cosh}\left\{(V-V_{\tau hNaP})/{\text{k}}_{\tau hNaP}\right\}.$$ (7)

In Eqs (4), (6) and (7), *V*_*xhNaP*_ and *k*_*xNaP*_ for *x* ∈ {*m*, *h*, *τ*) represent the gating variable’s half-activation voltage and slope, respectively. The values of all parameters used are provided in Table 1.

**Table 1:** Parameter values used in the model.

Membrane capacitance (pF)	*C* = 20.
Maximal conductances (nS)	${\bar{g}}_{NaP}=5$, ${\bar{g}}_{L}=2.8$, ${\bar{g}}_{SynE}=0.1$, ${\bar{g}}_{SynI}=0.6$
Reversal potentials (mV)	*E*_*Na*_ = 50, *E*_*LFO*_ = −63, *E*_*LEO*_ = −50, *E*_*SynE*_ = −10, *E*_*SynI*_ = −75
Synaptic weights	*a*_30_ = *a*_*v*0_ = 0.5, *a*_*D*_ = 0.35, *b*_1_ = 10, *b*_2_ = 0.1
Parameters of *f*(*V*) function (mV)	*V*_min_ = −50, *V*_max_ = 0
Parameters of *I*_*NaP*_ (mV)	*V*_*mNaP*_ = −40, *k*_*mNaP*_ = −6, *V*_*hNaP*_ = −55, *k*_*hNaP*_ = 10, *V*_*τNaP*_ = −40, *k*_*τNaP*_ = −12
Time constant (ms)	*τ*_*hNaP*_ = 4000
Rates of increase of leak reversal potentials and synaptic weights with respect to *α*	*β*_*E*_ = 0.1, *β*_*a*_ = 3

The output of each neuron (center) describing its activity (normalized firing rate) is represented by a piecewise linear function *f*(*V*), changing between 0 and 1 such that:

$$f(V)=\{\begin{array}{c} 0,\;\text{if}\;V<V_{\text{min}}\\ (\text{V}-{\text{V}}_{\text{min}})/(V_{\text{max}}-V_{\text{min}}),\;\text{if}\;V_{\text{min}}\leq V<V_{\text{max}}\\ 1,\;\text{if}\;V\geq V_{\text{max}} \end{array}$$ (8)

where *V*_min_ and *V*_max_ define the voltages at which threshold and saturation are reached, respectively.

**Fig 2:**
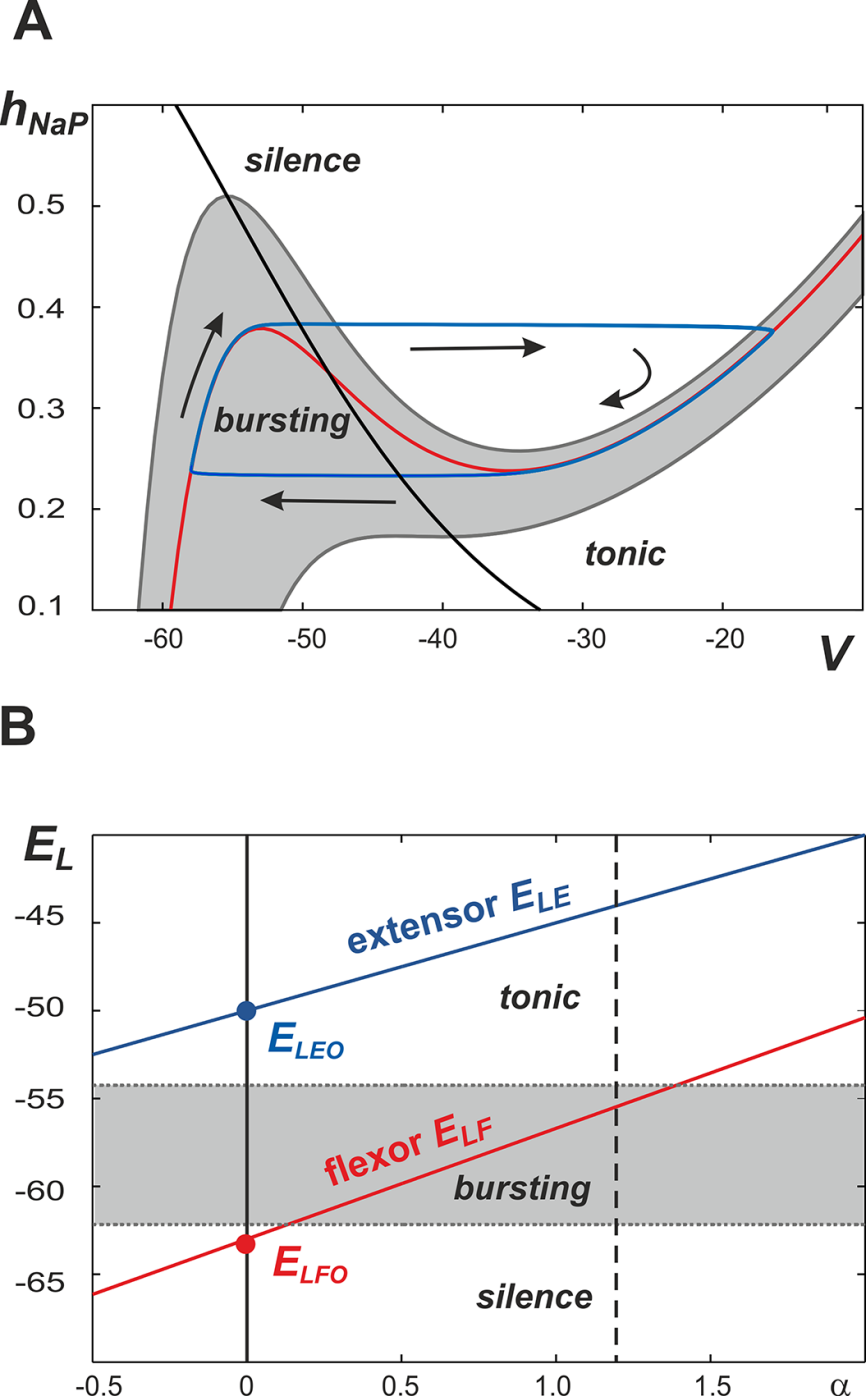
Operating regimes of single center and generation of rhythmic bursting. **A.** Representation of single center dynamics (blue line) in the (*V*, *h*_*NaP*_) plane. The *V*-nullclines with varying *E*_*L*_ values are represented by the gray band and the red line; the *h*_*NaP*_ -nullcline is shown by a black line. Bursting occurs when the value of *E*_*L*_ is such that the *V*-nullcline exists within the gray region. Silence and tonic behaviors occur at values of *E*_*L*_ that place the *V*-nullcline above and below the gray region, respectively. **B.** It is suggested that neuronal excitability is defined by the leak reversal potential, *E*_*L*_, which linearly increases with the parameter *α* representing NMDA concentration. Bursting occurs when *E*_*L*_ is greater than -62.7 mV and less than -54.2 mV (gray area). Silence and tonic behaviors are observed at values below and above this range, respectively. It is suggested that the basal value of *E*_*L*_ (at *α* = 0) for the flexor centers (*E*_*LFO*_) is below the bursting area, so that with an increase of *α* the flexor centers can generate bursting once the flexor *E*_*LF*_ is in the gray area. In contrast, the basal value of *E*_*L*_ for the extensor centers (*E*_*LEO*_) is much higher and these centers, if disconnected, operate in a mode of tonic activity. A dashed line at *α* = 1.2 denotes the maximal *α* value used in our simulations.

The persistent (slowly inactivating) sodium current, *I*_*NaP*_, provides a neuron with intrinsic rhythmicity. The behavior of this neuron can be considered using a (*V*, *h*_*NaP*_) phase plane (Fig 2A). The large time constant of *I*_*NaP*_ inactivation makes the time scales of the two dynamical variables, *V* and *h*_*NaP*_, substantially different which allows us to apply the fast-slow decomposition technique for qualitative analysis of neuronal behavior.

In the phase plane shown in Fig 2A, *V*- and *h*_*NaP*_-nullclines (red and black, respectively) are calculated by setting the right hand side of Eqs (1) and (6) equal to zero. *E*_*L*_ was increased to mimic higher levels of excitation and this was reflected by a downward movement of the *V*-nullclines on the phase plane. The band of nullclines where intrinsic bursting activity occurs is shaded gray. Within this area the *h*_*NaP*_-nullcline intersects with the *V*-nullcline at a point between the *V*-nullcline knees. This corresponds to an unstable critical point and allows for the emergence of a stable limit cycle. This limit cycle is shown in the (*V*, *h*_*NaP*_) phase plane by a neuron’s trajectory with the direction indicated by arrows. The neuron’s image point moves slowly along the left and right segments of the *V*-nullcline (slow timescale, silent and active phases) and quickly jumps between them (fast timescale, switching between phases) showing the behavior typical for a relaxation oscillator. When a neuron is silent its image point travels up the left branch of the *V*-nullcline until reaching the left knee causing it to jump toward the right branch of the *V*-nullcline in a movement representing activation of a neuron. Once the neuron is active, the corresponding trajectory will travel down the right branch of the *V*-nullcline until it reaches the right knee where it will then jump back to the left branch and the neuron will once again be silent.

Based on the above description, each isolated neuron can be in one of three regimes: silent, bursting (oscillatory), and tonic (constant activity) (Fig 2A) which depends on the level of neuronal excitation that, in the case of an isolated neuron, is defined by the leak reversal potential, *E*_*L*_. As shown in Fig 2B, bursting in the isolated neuron occurs when *E*_*L*_ ∈ (−62.7, −54.2) mV (gray area). When *E*_*L*_ is less than -62.7 mV, the *h*_*NaP*_-nullcline in Fig 2A intersects with the *V*-nullcline at a position to the left of its left knee (all nullclines that exist in the area marked "silence"). This creates a stable critical point on the inactive (left) branch of the *V*-nullcline where the neuron's position represents an inactive (silent) state. When *E*_*L*_ exceeds -54.2 mV, the *V*-nullcline in Fig 2A loses its cubic-like shape. This creates a stable critical point on the *V*-nullcline (all nullclines that exist in the area marked "tonic" in Fig 2A) and the neuron therefore demonstrates a constant level of activity, which is referred to as tonically active. Therefore, with an increase of excitation defined by *E*_*L*_ the neuron can potentially go through silent to bursting and then to tonically active regimes.

The model considered here consists of two (left, L, and right, R) bilaterally connected rhythm generators, each of which represents one flexor (F) and extensor (E) center (Fig 1). As described above, each center can be in silent, bursting, or tonic regimes, depending on the level of its excitation. However, we assume that, at least in the conditions of rhythmic bursting (fictive locomotion) evoked in the isolated rodent spinal cord by neuroactive drugs, the rhythm generators are asymmetric so that only the flexor centers (left, LF, and right, RF) operate in the bursting mode, whereas the extensor centers (left, LE, and right, RE), if isolated, operate in the regime of tonic activity (this issue is specifically addressed in the Discussion).

The excitabilities of the flexor and extensor centers (defined by their leak reversal potentials, *E*_*LF*_ and *E*_*LE*_, respectively) in the model are regulated by a parameter *α*. By introducing this parameter we did not intend to perform a biologically realistic simulation of the cellular mechanisms responsible for NMDA-induced bursting in spinal interneurons, which are not well understood. Instead, this parameter was used to simulate the general effect of NMDA, whose application in the isolated spinal cord preparations leads to an increase in neuronal activity and locomotor frequency [37,40].

To simulate the effect of the drug on the excitabilities of both centers, we suggest that:

$$E_{LF}=E_{LFO}\cdot (1-\beta_{E}\cdot \alpha );$$ (9)

$$E_{LE}=E_{LEO}\cdot (1-\beta_{E_{}}\cdot \alpha ),$$ (10)

where *E*_*LFO*_ and *E*_*LEO*_ define the basal values of the leak reversal potentials of the corresponding centers (at *α* = 0), and *β*_*E*_ defines the rate of their increase with *α*. To introduce the asymmetry suggested above, we set *E*_*LFO*_ close to the border between silence and bursting and *E*_*LEO*_ slightly above the bursting area (Fig 2B) so that with increase of *α* (in the uncoupled case), the flexor centers would always operate in the bursting mode, whereas the extensor center would be in the tonically active regime.

The bilaterally symmetrical network of four interacting centers with their interconnections (Fig 1B) can be described as follows:

$$C\cdot {\dot{V}}_{i}=-I_{NaPi}-I_{Li}-I_{SynEi}-I_{SynIi};$$ (11)

$$I_{NaP}={\bar{g}}_{NaP}\cdot m_{NaP\infty }(V)\cdot h_{NaP}\cdot (V-E_{Na});$$ (12)

$$I_{Li}={\bar{g}}_{L}\cdot (V_{i}\;-E_{Li}),$$ (13)

where *i* (*i* = 1,2,3,4) is the index corresponding to the center’s number as shown in Fig 1B. Currents *I*_*SynEi*_ and *I*_*SynIi*_ define, respectively, the excitatory and inhibitory synaptic interactions between the centers:

$$I_{SynE1}=(a_{3}\cdot f(V_{3})+a_{V}\cdot f(V_{4}))\cdot {\bar{g}}_{SynE}\cdot (V_{1}-E_{SynE});$$ (14)

$$I_{SynE3}=(a_{3}\cdot f(V_{1})+a_{V}\cdot f(V_{2}))\cdot {\bar{g}}_{SynE}\cdot (V_{3}-E_{SynE});$$ (15)

$$I_{SynI1}=(a_{D}\cdot f(V_{3})+b_{2}\cdot f(V_{2}))\cdot {\bar{g}}_{SynI}\cdot (V_{1}-E_{SynI});$$ (16)

$$I_{SynI2}=b_{1}\cdot f(V_{1})\cdot {\bar{g}}_{SynI}\cdot (V_{2}-E_{SynI});$$ (17)

$$I_{SynI3}=(a_{D}\cdot f(V_{1})+b_{2}\cdot f(V_{4}))\cdot {\bar{g}}_{SynI}\cdot (V_{3}-E_{SynI});$$ (18)

$$I_{SynI4}=b_{1}\cdot f(V_{3})\cdot {\bar{g}}_{SynI}\cdot (V_{4}-E_{SynI}),$$ (19)

where ${\bar{g}}_{SynE}$ and ${\bar{g}}_{SynI}$ are the maximal conductances of excitatory and inhibitory synaptic channels, *E*_*SynE*_ and *E*_*SynI*_ are the reversal potentials of these channels, and *a*_*D*_, *a*_*V*_, *a*_3_, *b*_1_, and *b*_2_ define the synaptic weights of, respectively, V0_D_, V0_V_, V3, In1, and In2 inputs to the centers in accordance with their interactions shown in Fig 2B.

An increase in NMDA in the spinal cord preparation affects not only the level of excitation of neurons representing the centers, but also the level of excitation and recruitment of CINs. To take this into account in our simplified model, in which CINs are not explicitly included and the pathways they mediate are replaced by direct synaptic connections (see Fig 1B vs. 1A), we need to make synaptic weights of these connections to increase with the NMDA concentration represented by the parameter *α*. This especially concerns the excitatory commissural pathways that are amplified by neuron recruitment via excitatory interactions within each population and may also involve other excitatory neurons, such as the V2a, which are also recruited and activated with an increase in the NMDA concentration and locomotor speed and mediate inputs to the excitatory V0v CINs [37,57,58]. In other words, the increase of synaptic weights of CIN pathways was included in our simplified model to compensate for removal of CIN neurons whose excitability should also be affected by the drug. Therefore, we suggested that:

$$a_{3}=a_{3O}\cdot (1+\beta_{a}\cdot \alpha )\;;$$ (20)

$$a_{V}=a_{VO}\cdot (1+\beta_{a}\cdot \alpha )\;,$$ (21)

where *a*_*VO*_ and *a*_3*O*_ define the basal weights of the corresponding excitatory connections and *β*_*a*_ defines the rate of their increase with *α*.

All model parameters are specified in Table 1. Synaptic weights were first set by manual adjustment to match experimental recordings. Weights were then optimized by running several iterations of one-dimensional bifurcation diagrams (see next section), where the robustness of a given regime was assessed by the width of the parameter range across which it displayed proper behavior, i.e. matching experimental phase diagrams and flexor-flexor phase relationships. Simulations were performed using custom C^++^ programs and visualized using gnuplot.

### Bifurcation Diagrams

To inspect changes in the model behavior with changing neuronal excitation (defined by the parameter *α*), phase differences between the left (LF) and right (RF) flexor activities or between ipsilateral flexor (LF) and extensor (LE) activities were calculated as the time differences between onsets of the corresponding bursts divided by the oscillation period measured in the activity of the flexor center. In the chosen range, *α* ∈ [0, 1.2] was progressively increased or decreased in a step-wise manner with a step size comprising 1/1000 of the range. At each point, the simulation was run and the phase differences were computed. Moreover, at every time step the initial conditions were chosen as a final state of the system from the previous time step to minimize a transient period. After convergence of the phase difference to a steady state with preset accuracy its value was plotted versus the parameter value. For each bifurcation diagram two series were generated: one with *α* increasing from 0 to 1.2 in the described manner and another with *α* decreasing from 1.2 to 0. The qualitative changes in system behavior (bifurcations) can be seen on such diagrams as jumps, corners, branching and other types of discontinuities. In addition, the existence of non-overlapping branches obtained by changing the parameter in different directions serves as an unequivocal indication of bi- or multi-stability in the system in the corresponding parameter ranges.

In addition to the phase differences, the values of amplitude and instantaneous frequency (reciprocal to the oscillation period) were also calculated and the corresponding plots were built. Furthermore, the frequency of flexor activity was used to construct diagrams where phase differences between left (LF) and right (RF) flexors were plotted against the frequency.

### Experimental Data

The experimental data included for comparison with modeling results had been obtained in earlier experimental studies published in Nature [37]. No new animal data were collected. All experiments were approved by the local ethical committee and performed in accordance with European guidelines for the care and use of laboratory animals. Briefly, the experiments were performed in the isolated spinal cords of wild-type (control) and transgenic mice with ablated V0_D_ or V0_V_ or both V0 neuron types (the detailed description of the transgenic lines of mice used can be found in [37]. Mice aged E18.5 (with genetically deleted V0_D_ neurons) or newborn mice aged 1–2 days (for all other studies) were used. The isolated spinal cords in a chamber were perfused with normal Ringer’s solution. Locomotor-like activity was induced by the exogenous application of mixtures of serotonin (or 5-hydroxytryptamine, 5-HT) and NMDA (N-methyl-D-aspartate). The locomotor frequency was controlled by concentrations of locomotor-inducing drugs (mostly NMDA) [37,40]. All recordings were performed at room temperature (22–24°C). Locomotor activity was recorded with suction electrodes attached to the L2 and L5 lumbar roots on both sides of the cord. The raw activity was band-pass filtered at 100 Hz to 1 kHz. Data points for analyzing cycle periods and phases were taken after the locomotor activity had stabilized 10–15 min after the initial burst of activity. All details of recordings and data processing can be found in [37].

## Results

### Performance of the Intact Network: Left-Right Alternation at Any Excitation Level

**Fig 3:**
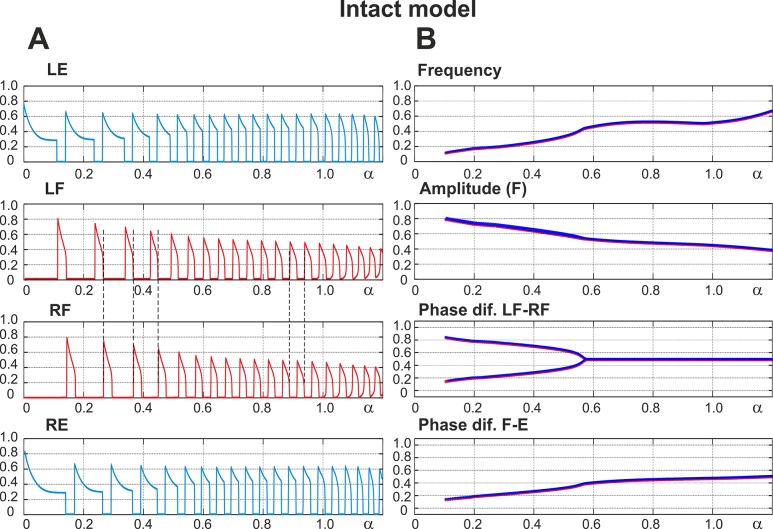
Performance of the intact model. **A.** Changes in the output activity of the four centers with increasing neuronal excitation (*α*). **LE** and **RE** indicate left and right extensor centers, respectively; **LF** and **RF** indicate left and right flexor centers. For low values of *α* (*α* < 0.55) RF activates upon LF shutdown (see vertical dashed lines). Due to asymmetry of flexor and extensor phase duration at low values of *α* there is a part of each step cycle when both flexor centers are inactive and hence both extensor centers are active. *α* was changed linearly in time from *α* = 0 at t = 0 to *α* = 1.2 at t = 120 s. **B.** Bifurcation diagrams for different dynamical characteristics. From top to bottom: **Frequency**—frequency of bursts in Hz; **Amplitude (F)**—flexor center amplitude (maximum output activity of flexor center); **Phase dif. LF-RF**—phase difference between left and right flexor centers; **Phase dif. F-E**—phase difference between ipsilateral flexor and the extensor centers. When *α* increases there is an increase in frequency (mostly concerned with shortening of extensor phase) as well as a decrease in the amplitude of each flexor center burst (see also **LF** and **RF** panels on the left). For relatively high values of excitation (*α* > 0.55) the phase difference between flexor centers is exactly 0.5. This means that the **RF** activates exactly one half-period after the **LF**. At lower values of *α* two distinct regimes exist: the first has a phase difference between flexor centers less than 0.5 (lower branch) which corresponds to the activation pattern shown in **A** (LF leads, RF activates on LF’s shutdown, then a pause until LF activates again) and the second regime has a phase difference greater than 0.5 (upper branch) when RF activates first and LF activates on RF’s shutdown (not shown on **A**). Note that in all diagrams in **B**, *α* was changed in both directions, first forward from 0 to 1.2 and then backward from 1.2 to 0. However, in all diagrams in Fig 3B, the graphs for forward (red) and backward (blue on the top) changes of *α* overlapped. In summary, the intact model demonstrates left-right alternating activity at all levels of excitation (all values of *α*).

The performance of the intact network is shown in Fig 3. Panel A of the figure shows the changes in the output activity, *f*(*V*), of all four centers with *α* changing from 0 to 1.2. The vertical dashed lines in this panel indicate that activities of left (LF) and right (RF) flexor centers alternate at all values of *α*. Panel B shows how the frequency of oscillations (top diagram), the amplitude of flexor activity (second diagram), and the phase differences between the activities of left and right flexor centers (LF-RF) and left flexor and left extensor centers (F-E) (two bottom diagrams) changed with *α*. Note that in contrast to panel A, panel B shows *α* being changed in both directions, first forward from 0 to 1.2 (red) and then backward from 1.2 to 0 (blue). However, in all diagrams in Fig 3B, the graphs for forward and backward changes of *α* fully overlapped.

Fig 3A and two top diagrams in Fig 3B show that with increasing *α* (simulating an increase of neuronal excitation in the experimental preparations by the administration of NMDA) the amplitude of flexor center activity in the model monotonically decreases and the locomotor frequency monotonically increases, which generally fits experimental data [40]. The bifurcation diagram depicting the phase difference between flexor centers in the intact model (Fig 3B, Phase diff. LF-RF) shows two intervals where the model exhibits qualitatively different behavior. On the right (*α* > 0.55) flexor centers demonstrate perfect anti-phase synchronization (Δ*φ* = 0.5). This represents a symmetric alternation between the activities of left and right flexor centers that is in contrast to the behavior seen at lower values of *α* (*α* > 0.55) where the phase difference between the alternating flexor center activities is not 0.5 and a pitchfork bifurcation occurs at *α* ≈ 0.55. The branches of the pitchfork reflect direct inhibitory interactions between the flexor centers (rebound) and differ depending on which left or right flexor center activates first. Moreover, the supercritical pitchfork bifurcation results in instability of the symmetric anti-phase regime with a decrease in *α*. The existence of two symmetric branches both emerging from Δ*φ* = 0.5, one with Δ*φ* > 0.5 and the other with Δ*φ* < 0.5, is concerned with the symmetry of the underlying network. It is worth mentioning that a slightly non-symmetric network would favor one of these two branches and weaken the other. In this case, the pitchfork bifurcation would be replaced with a saddle-node bifurcation resulting in the emergence of the “weak” branch, while the “strong” branch would replace the perfect anti-phase regime. Accordingly, the major qualitative change in the system dynamics as *α* is decreased below the bifurcation value of *α* ≈ 0.55 is concerned with the appearance of two distinct stable regimes with phase shift between flexor center activities greater and less than 0.5.

It is important to note that at high values of *α* the periods of flexor and extensor activity have the same duration, while at low values of *α* the flexor phases can be significantly shorter than the extensor ones (see left parts of the graphs in Fig 3A). However, although the activity of left and right flexor centers in this case is not symmetric (does not alternate in perfect anti-phase), the left and right flexor centers are never active at the same time. Therefore, at any value of *α* (and at any locomotor frequency, see the top diagram in Fig 3B), the intact network exhibits left-right alternation of flexor activity.

### Removal of All V0 Commissural Pathways: Left-Right Synchronization at Any Level of Excitation

**Fig 4:**
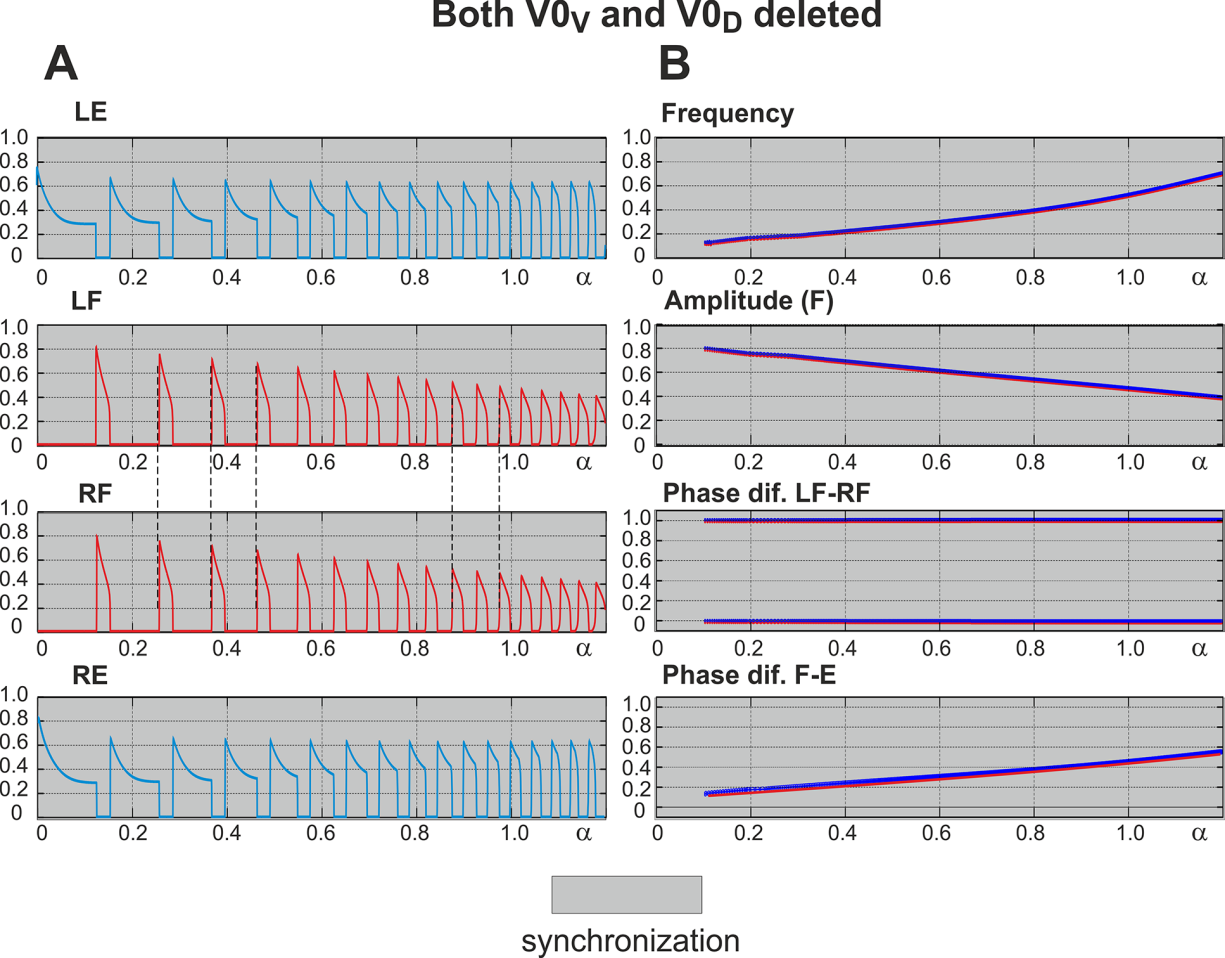
Performance of the model with both V0 pathways removed. **A.** Changes in the activity of the four centers with increasing neuronal excitation (*α*). Mutual excitation between **LF** and **RF** maintains left-right synchronized behavior as indicated by the dashed lines. **B.** Bifurcation diagrams. Frequency and amplitude of flexor center oscillations change similarly to the intact case (see Fig 3 for comparison). The phase difference between LF and RF is 0 or 1, indicating perfect left-right synchronization for all values of *α*. Other details and explanations can be found in the legend to Fig 3. As in the intact case, *α* was changed in both directions, first forward from 0 to 1.2 (red) and then backward from 1.2 to 0 (blue on the top), but graphs for forward and backward changes of *α* overlap. In summary, when both V0 pathways are removed, the model exhibits left-right synchronized “hopping” behavior at all levels of excitation (all values of *α*).

After both V0 commissural connections are eliminated, the interactions between left and right centers are provided exclusively by the excitatory V3 pathways (see Fig 1). As shown in Fig 4 (panel A and two top diagrams in panel B), similar to the intact model the amplitude of flexor activity monotonically decreases with increasing *α*, whereas the locomotor frequency monotonically increases. In contrast to the intact case, the activities of left and right centers are fully synchronized at all values of *α*. This is seen in Fig 4, panel A (indicated by vertical dashed lines) and panel B (the phase difference between the activities of left and right flexor centers remains 0 or 1 for all values of *α* - see “Phase dif. LF-RF” diagram). This left-right synchronous activity corresponds to hopping behavior.

### Selective Removal of V0_V_ Commissural Pathways: Switching from Left-Right Alternation to Left-Right Synchronization with Increasing Excitation

**Fig 5:**
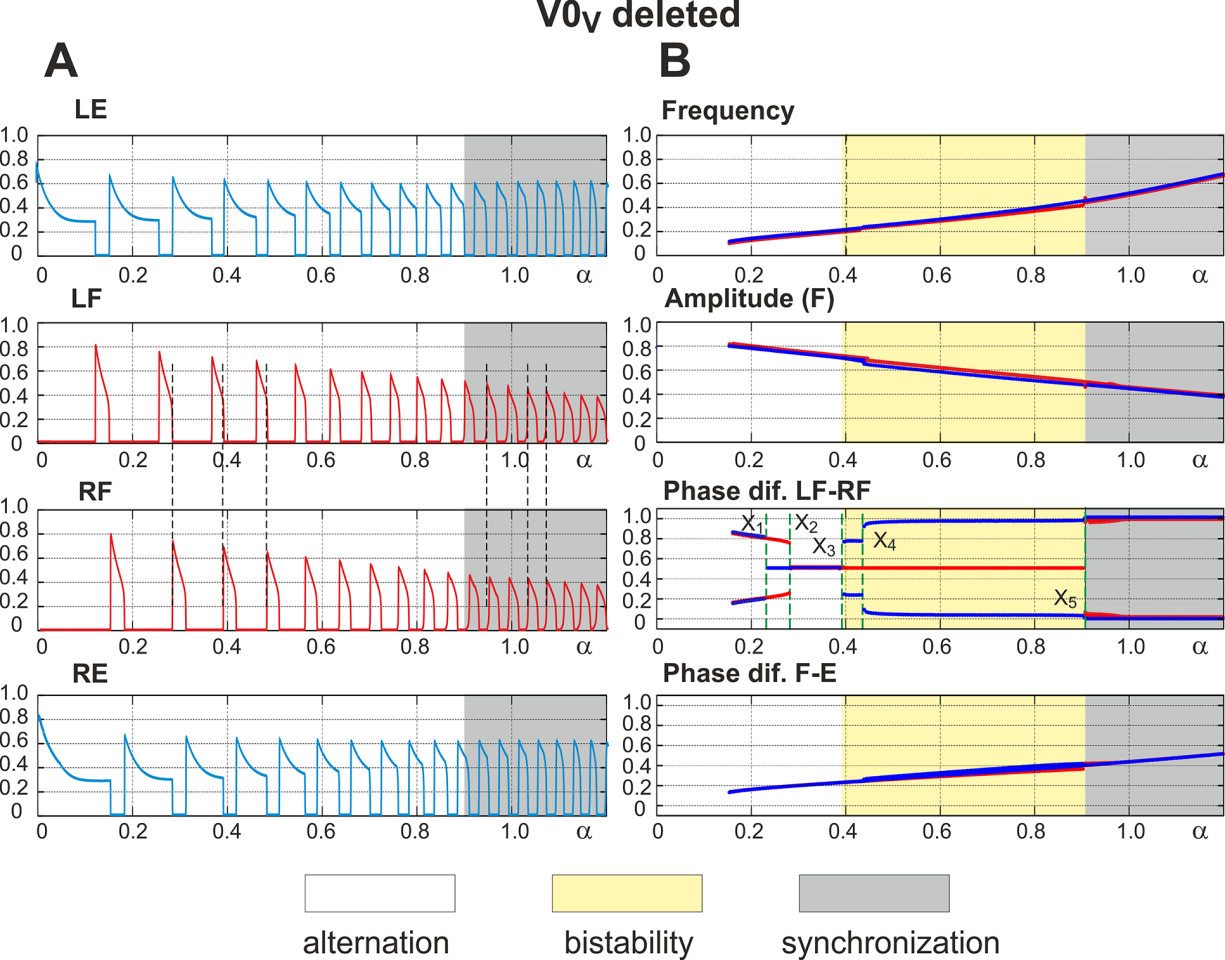
Performance of the model with the excitatory V0_V_ pathways removed. **A.** Changes in the activity of the four centers with increasing neuronal excitation (*α*). Vertical dashed lines indicate left-right alternation of flexor center activity at low values of *α* and its synchronization at *α* > 0.9. **B.** Bifurcation diagrams. Frequency and amplitude of flexor center oscillations change similar to the intact case (see Fig 3 for comparison). *α* was changed in both directions, first forward from 0 to 1.2 (red lines) and then backward from 1.2 to 0 (blue lines). The non-overlapping branches indicate bistability. A series of such bistable regions exist in the diagram showing phase differences between the flexor centers (**Phase dif. LF-RF**). They are described in the text. In general, at low values of *α* (*α* < X_3_ ≈ 0.39), we have different forms of left-right alternation (see text for details), at *α* < X_5_ = 0.9 there is a perfect left-right synchronization, and in the middle (X_3_ < *α* < X_5_) the system exhibits a bistable behavior.

Selective deletion of the V0_V_ subtype of V0 CINs corresponds, in our model, to elimination of V0_V_ excitatory connections from extensor centers to the contralateral flexor centers (see Fig 1). In this case, the left and right sub-networks interact only through the excitatory (V3) and inhibitory (V0_D_) pathways between the flexor centers. Independent of commissural connections and similar to the intact case, the amplitude of flexor activity monotonically decreases with increasing *α*, whereas the locomotor frequency monotonically increases (see Fig 5, panel A and two top diagrams in panel B). Depending on the balance between the excitatory V3 and inhibitory V0_D_ pathways, the net interactions can be excitatory (if the excitatory connections prevail) or inhibitory (if mutual inhibition is stronger than excitation).

In our model, a pharmacologically-induced increase in excitation is implemented as an increase in the leak reversal potentials in the centers as well as an increase in the weights of excitatory pathways (to account for drug effects on neurons involved in these pathways). Based on our suggestions, the model parameters are set in such a way that at low levels of excitation (at low values of *α*) the inhibitory connections between flexor centers dominate, while at high excitation (at high values of *α*) the net interaction becomes excitatory. As a result, the model demonstrates left-right alternation of flexor activity at low values of *α* and left-right synchronization at high values of *α* (both indicated by vertical dashed lines in Fig 5A). The bifurcation diagram in Fig 5B (see “Phase dif. LF-RF” diagram) shows that at extremely low levels of excitation (*α* < X_1_) the activities of flexor centers alternate similarly to the intact case (compare the two branches left of X_1_ in “Phase dif. LF-RF” diagram in Fig 5B with the corresponding diagram in Fig 3B). At very high excitation levels (*α* > X_5_) left-right synchronous activity occurs similarly to the case when all V0 pathways are removed (compare two blue branches in the right part of “Phase dif. LF-RF” diagram in Fig 5B with the corresponding panel in Fig 4B). For intermediate excitation levels (X_1_ < *α* < X_5_) the system demonstrates a complex transition scenario from alternating to synchronous activity that is explained below using the technique of fast-slow decomposition.

**Fig 6:**
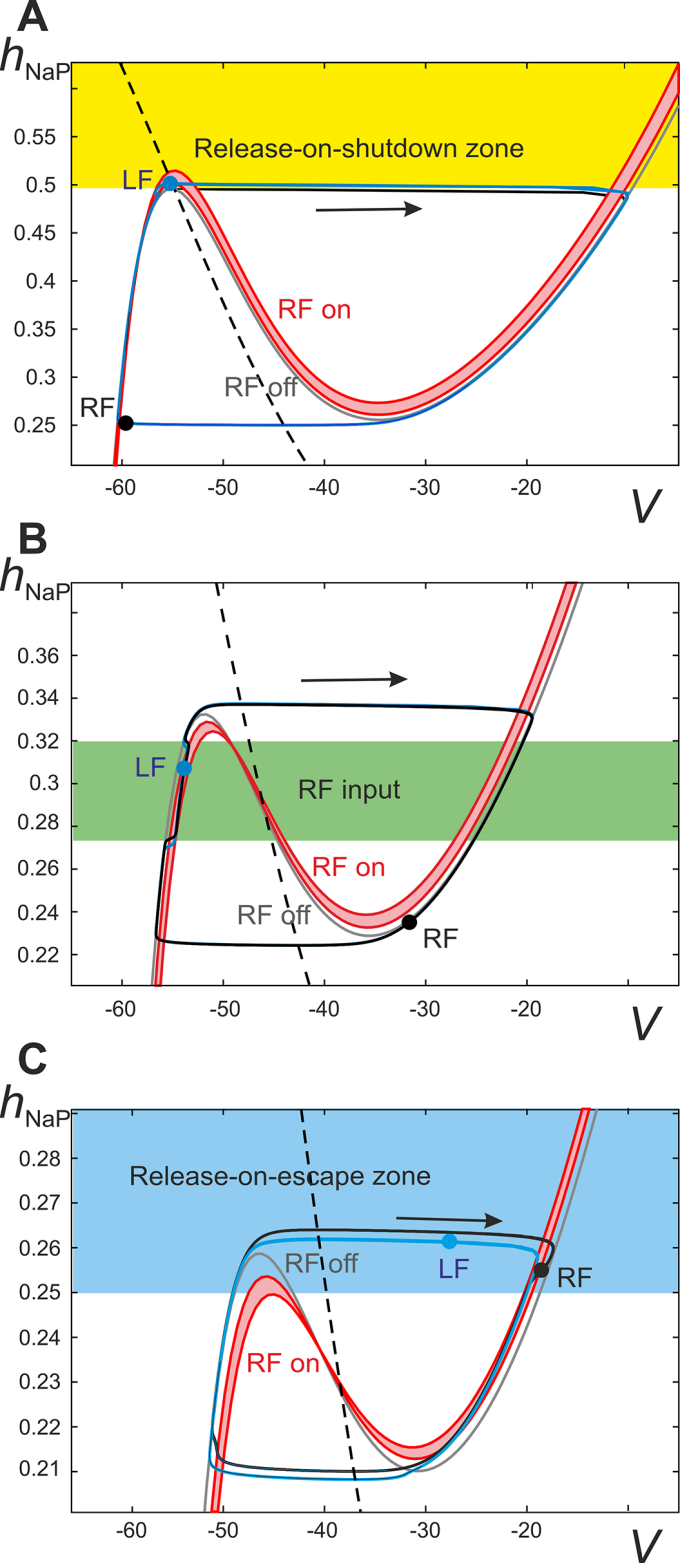
Fast-slow decomposition of the model dynamics when the V0_V_ excitatory pathways are deleted. The trajectories of left and right flexor centers (LF and RF) are projected onto the (*V*, *h*_*NaP*_) plane (blue and black curves, respectively). The red nullcline band (RF on) depicts the range of input received by the left flexor center from the right flexor center during the activity period of the latter. Due to a gradual decrease of activity in the right flexor center during its active phase the red nullcline moves upward slightly during the RF burst and therefore shades the red area. The solid gray cubic-like curve (RF off) represents a *V*-nullcline for an uncoupled flexor center (i.e. receiving no inputs), and the dashed black line represents the nullcline for the slow variable, *h*_*NaP*_. Sample positions, or image points, of flexor centers are depicted by the black (RF) and blue (LF) circles. Nullclines depicting inputs from extensor centers are absent because there are no commissural extensor-to-flexor connections since V0_V_ connections are deleted. **A.** Starting at any initial conditions in the yellow area (i.e. with *h*_*NaP*_ higher than the left knee of the gray *V*-nullcline) and being uncoupled the LF will immediately activate. When *α* < X_2_ in “Phase dif. LF-RF**"** diagram in Fig 5B, the left knees of the red nullclines are at higher *h*_*NaP*_ values than the left knee of the unperturbed (gray) *V*-nullcline (i.e. in the yellow area) thus making it possible for the LF to reach the yellow area during the active phase of the RF (i.e. by climbing up in the red band). Once the LF is in the yellow area, the deactivation of RF satisfies the conditions for immediate LF activation by releasing it from inhibition (release-on-shutdown, see text). Further, due to a relatively long extensor phase, at low *α* the image point for RF moves up too slowly after its deactivation and thus cannot reach the yellow area before the LF shuts down. Accordingly, there is a phase in the step cycle in which both flexors are inactive and therefore climbing up along the left branch of the gray *V*-nullcline until the RF escapes. This regime corresponds to the alternation of flexor center activities with a phase difference Δ*φ* ≠ 0.5 (“coupled” alternations, see Fig 5A)**. B.** When *α* > X_4_ the left knees of the red *V*-nullclines are at lower *h*_*NaP*_-coordinates than the gray *V*-nullcline (indicating a state of mutual excitation) thus preventing the release-on-shutdown mechanism described above. Despite the presence of mutual excitation between flexor centers alternation can occur when the flexor centers are initialized in opposing phases, i.e. alternation (see text for more detailed explanations of the mechanism). When the RF is active it causes a slight rightward perturbation of LF (area highlighted in green). **C.** Using the same *α* value as in panel **B**, the synchronization of flexor activity may be achieved when initial conditions favor hopping, i.e. initial conditions for the LF within the region highlighted in blue, above the left knee of the red nullclines (labeled as RF on), will lead to immediate LF activation once the RF is active. If both flexor centers are inactive (i.e. their image points are on the left branch of the gray *V*-nullcline) and they have similar positions in (*V*, *h*_*NaP*_) with the RF slightly ahead (i.e. at higher values of *h*_*NaP*_), then at the time of RF escape the LF is in the blue zone and hence it immediately activates as well. This leads to synchronization of the flexor centers via the release-on-escape mechanism (see text for details).

As seen in Fig 2A, the left knee on the *V*-nullcline is affected by changes in excitation to a much greater extent than the right knee (compare, for example, the width of the shaded area in terms of *h*_*NaP*_ values at *V* = –55mV and at *V* = –30mV). Therefore, the activity of each flexor center can be affected by a synaptic input during a period of its inactivity, i.e. during the ipsilateral extensor phase, more efficiently than during its active phase. Due to intrinsic bursting properties each flexor center is capable of endogenous escape and inactivation. Accordingly, two types of strong synchronizing events are possible: a release of one flexor center on an escape of the other (*release-on-escape*) and a release of one flexor center when the other deactivates (*release-on-shutdown*). The former mechanism creates (in-phase) synchronization between flexor centers (“hopping”), while the latter underlies their alternation (anti-phase synchronization). Fig 6A–6C illustrates and allows for a more comprehensive understanding of system behavior after V0_V_ pathways are deleted.

#### Low excitation and inhibitory interaction domination: Release-on-shutdown

As mentioned above, when the level of excitation is very low (*α* < X_1_ in “Phase dif. LF-RF” diagram in Fig 5B), the net interactions between flexor-centers are inhibitory (V0_D_ pathways dominate over V3 ones). Fig 6A shows the phase portrait in this case. The net inhibition manifests itself by the position of the flexor-to-flexor (red) *V*-nullclines relative to the gray nullcline representing lack of interactions; specifically, the left knees of the red nullclines exist at higher *h*_*NaP*_-coordinates than the one of the gray nullcline. This represents a state of lower excitation characterized by net inhibition between flexor centers. In order for left-right alternation to occur in a state of net flexor-to-flexor inhibition, a release-on-shutdown must happen when the activity of the currently active (leading) flexor center terminates. The lagging flexor center is inhibited when the leading flexor center is active and the lagging flexor center must therefore rise to an *h*_*NaP*_-coordinate above the left knee of the gray *V*-nullcline when the activity of the leading flexor center terminates. Following the activation of the lagging flexor center and its subsequent termination, both flexor centers remain inactive until the leading flexor center activates again. Because of this period of inactivity of both flexor centers, the left-right alternation between flexor centers occurs at a phase difference Δ*φ* ≠ 0.5.

#### Transition to net excitatory interactions and exact anti-phase synchronization

In “Phase dif. LF-RF” diagram in Fig 5B, the X_2_ line indicates the transition from a state of net flexor-to-flexor inhibition to a state of net flexor-to-flexor excitation. This corresponds to the diagram shown in Fig 6B. This figure shows that the left knees of the red nullclines now exist at lower *h*_*NaP*_-coordinates than the gray nullcline. Fig 5B shows that as *α* increases the phase difference between flexor center activities eventually becomes exactly Δ*φ* = 0.5 (at *α* < X_2_). At this point the duration of the flexor phase is still much shorter than the extensor phase. The flexor-extensor phase difference corresponds to the flexor duty cycle (which is about 0.2, see the lowest panel in Fig 5B). This creates the prerequisites (short duty cycle together with weak reciprocal excitation) for the mechanism underlying the symmetric alternation as explained below.

When excitation is increased beyond X_2_ (in “Phase dif. LF-RF” diagram in Fig 5B) net flexor-to-flexor interaction is excitatory. This moves the red (flexor-to-flexor) *V*-nullclines in Fig 6B below the gray nullcline, representing a lack of interactions, and no longer allows for the release-on-shutdown mechanism of center alternation. Despite the net excitation between flexor centers, the left-right alternation is preserved within a range of increasing excitation (up to X_5_ on Fig 5B). Due to the aforementioned flexor-extensor asymmetry resulting from a relatively short duration of flexor activity, each flexor center receives inputs from the contralateral flexor center only during a short active phase of the latter. If the input comes during the inactive phase, and the recipient is far enough from the escape point at the left knee of its *V*-nullcline, thus preventing its immediate activation by release-on-escape, this input causes a slight depolarization shifting the trajectory to the right from the gray nullcline (see Fig 6B). When the contralateral flexor center deactivates, and the input is gone, the trajectory returns to the gray *V*-nullcline. When such a transient input occurs, the recovery of the inactivation variable *h*_*NaP*_ is slowed down because of a decrease in its steady state inactivation function *h*_*∞NaP*_ (*V*) which lowers the rate of change of *h*_*NaP*_ (see Eq (6)). Hence, a short excitatory input from the contralateral flexor center arriving during the inactive phase of the given center delays its activation.

The slope of the steady state inactivation function *h*_*∞NaP*_ (*V*) becomes steeper with the increasing voltage as the image point of a flexor center progresses along the left branch of the *V*-nullcline. So at higher voltages the delay in *h*_*NaP*_ recovery caused by the same transient voltage fluctuation is longer. Due to this fact the later the depolarizing stimulus arrives during the inactive phase of the flexor center (i.e. the smaller the time difference between the input and the activation of the flexor center), the more strongly the center activation is delayed.

Interactions between the flexor centers are reciprocal. If the first flexor center activates during the inactive phase of the second one, the activation of the latter is delayed. But once activated, the second flexor center in turn delays activation of the former. If the activation of the second flexor center occurs soon after deactivation of the first flexor center (i.e. with phase difference less than 0.5), then the first flexor center activates late in the second flexor center’s inactive phase (i.e. with phase difference greater than 0.5). Accordingly, the first center pushes the second back more strongly than the second pushes the first. Therefore, as the phase difference approaches one half of a step cycle, the flexor centers start pushing each other’s activation back with equal strength and hence the phase difference stabilizes at Δ*φ* = 0.5.

#### Intermediate to high excitation level: Release-on-escape and bistability

As the description above implies, weak reciprocal excitatory connections between flexor centers with sufficiently small duty cycles leads to bistability. Besides the regime of exact alternation which extends up to the line X_5_ in “Phase dif. LF-RF” diagram of Fig 5B, we also have stable synchronization between the flexor centers for excitation levels greater than X_4_. This means that depending on the model’s initial conditions we can expect stable left-right synchronization (hopping) or left-right alternation for a single value of *α* (this is illustrated in Fig 6C). The stability of synchronized flexor center oscillations is maintained by the release-on-escape mechanism. If the activity of the lagging flexor center reaches an *h*_*NaP*_-coordinate above the left knee of the *V*-nullcline, representing the maximal contralateral flexor center excitation (upper red curve in Fig 6C), the image point of the lagging flexor center immediately jumps up following the activation of the leading center. This decreases the phase difference between flexor center activations leading to their synchronous (or near synchronous) oscillations.

#### Bifurcation scenario

The overall bifurcation scenario in the case when V0_V_ pathways are removed is as follows (see “Phase dif. LF-RF” diagram in Fig 5B). Increasing *α* to the level of *α* = X_2_ causes the phase difference between flexor center activities to become exactly 0.5 and then remain at this value until *α* = X_5_. At this point the stable regime of flexor center alternation is destroyed due to step cycle shortening and the system switches to a regime with perfectly synchronized left and right flexor center activities (hopping). Importantly, as *α* decreases backward from a maximum value of 1.2, the system exhibits a wide hysteresis. The synchronization between flexor centers persists until *α* = X_4_, where a transition to alternation begins. First, the system switches to a regime with a relatively small phase difference between flexor centers which corresponds to overlapping left and right flexor bursts. This regime exists while *α* > X_3_ where it disappears and the system transitions to pure anti-phase flexor oscillations. Interestingly, as *α* further decreases, two new branches with phase difference greater and less than 0.5 appear due to a subcritical pitchfork bifurcation at *α* = X_1_ and not via a supercritical one as in the intact case. This implies that there is yet another bistability region between *α* = X_1_ and *α* = X_2_ where a “symmetric” left-right alternation in activity of flexor centers (with Δ*φ* = 0.5) coexists with “coupled” alternations (characterized by Δ*φ* < 0.5 and Δ*φ* > 0.5).

In summary, at levels of excitation less than X_3_ (*α* < X_3_) the model shows alternation of flexor center activities. When excitation exceeds X_5_ (*α* > X_5_) flexor centers demonstrate synchronous activity. Between these two bifurcations, i.e. X_3_ < *α* < X_5_, both regimes exist and are stable, and left-right phase relationships depend on initial conditions.

In summary, fast slow decomposition of system behavior reveals dynamical mechanisms responsible for the transition between different synchronization regimes as neuronal excitation increases. At low levels of excitation, flexor centers exhibit large burst amplitude (Fig 5B), which provides strong inhibitory interactions between these centers mediated by the inhibitory V0_D_ connections at relatively weak excitatory V3 connections. This results in strong net inhibitory interactions between left and right flexor centers. Hence, the inactive contralateral flexor center can be released from inhibition and become active only upon shutdown of the currently active flexor center. That explains how left-right alternation at low levels of excitation and low locomotor frequencies is provided by the V0_D_ commissural connections.

With an increase in excitation (*α*), we have two independent processes. First, the amplitude of flexor bursts is decreasing (Fig 5), and the mutual inhibition between flexor centers is reducing proportionally to the amplitude. Second, the excitatory V3 connections get stronger (see Eq (20)). Together these processes eventually lead to switching from net inhibition to net excitation (Fig 6B and 6C) and, via the release-on-escape mechanism (Fig 6C), to left-right synchronization.

At intermediate frequencies the system exhibits bistability with both in-phase and out-of-phase regimes stable. The stability of the latter is provided by net excitatory interactions between the flexor centers in combination with a relatively short flexor duty cycle (Figs 5B and 6B).

### Selective Removal of V0_D_ Commissural Pathways: Switching from Left-Right Synchronization to Left-Right Alternation with Increasing Excitation

**Fig 7:**
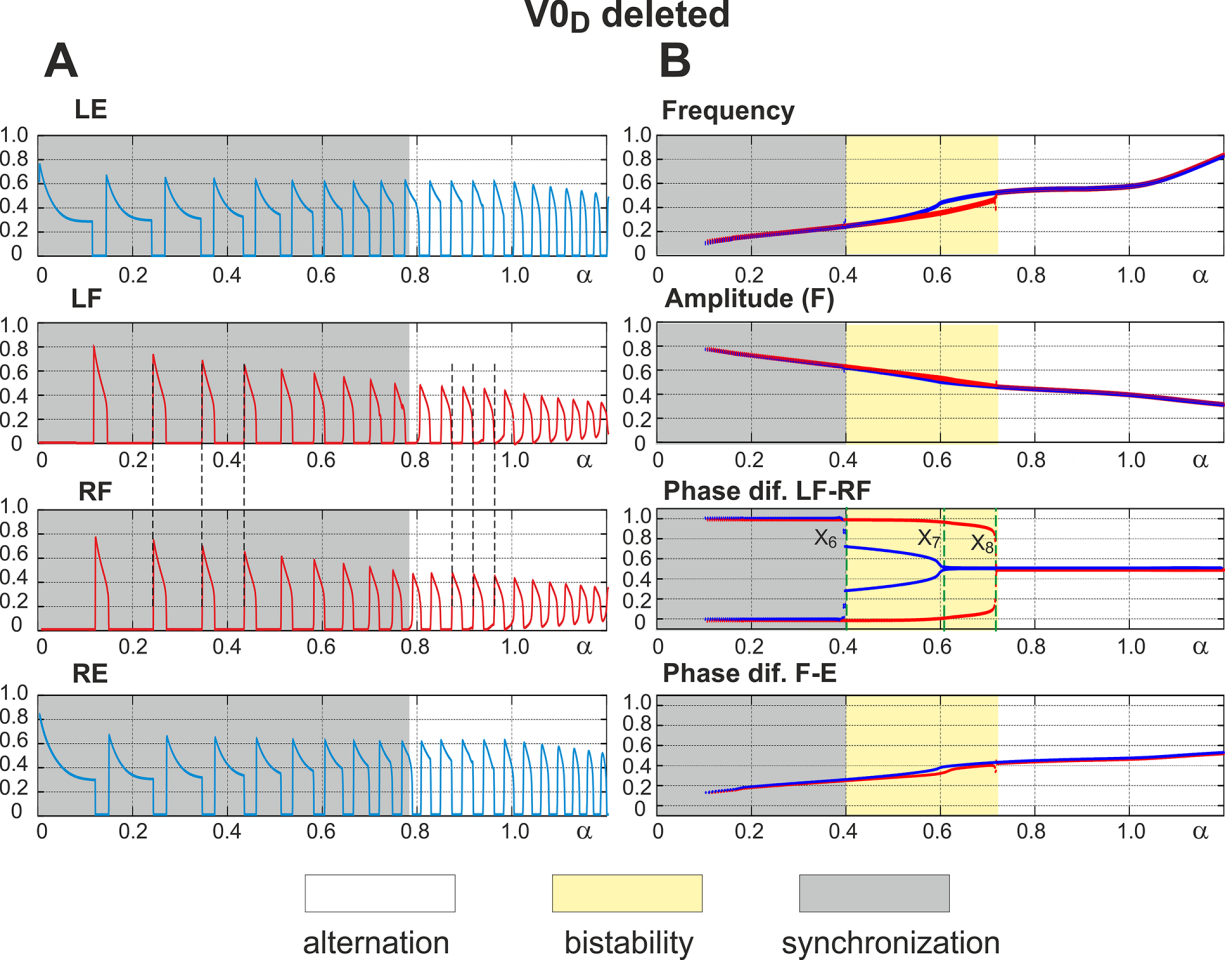
Performance of the model with the inhibitory V0_D_ pathways removed. **A.** Changes in the activity of the four centers with increasing neuronal excitation (*α*). Dashed lines are used to highlight LF-RF synchronization at low values of *α* and LF-RF alternation at *α* >0.8. **B.** Bifurcation diagrams. Frequency and amplitude of flexor center oscillations behave in a manner similar to the intact case (see Fig 3, for comparison). *α* was changed in both directions, first forward from 0 to 1.2 (red lines) and then backward from 1.2 to 0 (blue lines). Non-overlapping branches indicate bistability. The bifurcation diagram “**Phase dif. LF-RF**” has three bifurcation points, X_6_-X_8_. When *α* increases a single bifurcation occurs as LF-RF synchronization transitions to alternation at X_8_. Decreasing *α* causes two bifurcations: (i) transition from symmetric alternations of flexor activity, i.e. with a phase difference of Δ*φ* = 0.5, to alternations with a phase difference of Δ*φ* ≠0.5 at X_7_ and then (ii) from alternation to synchronization of flexor activity at X_6_. Hence, when the inhibitory V0_D_ pathways are removed the model demonstrates LF-RF synchronization that transitions to alternation as *α* increases.

Removal of V0_D_ corresponds to termination of reciprocal inhibition between the flexor centers (see Fig 1). Since V0_V_-dependent connections remain intact, the network has two competing mechanisms of left-right coordination. One of them concerns reciprocal excitation between the flexor centers provided by V3 connections, which tends to synchronize their activity. The other is characterized by excitation from contralateral extensor centers to each flexor center, which contributes to alternation of flexor center activities. The results of simulations in this case are shown in Fig 7A and 7B. Again, similar to the intact case, the amplitude of flexor activity monotonically decreases with increasing *α*, whereas the locomotor frequency monotonically increases (see panel A and two top diagrams in panel B). In contrast to the previous case, when V0_V_ pathways were removed, the model now demonstrates left-right synchronization of flexor center activity at low values of *α* and left-right alternation at high values of *α* (indicated by vertical dashed lines in Fig 7A). The transition scenario from synchronization to alternation is best seen in “Phase dif. LF-RF” diagram in Fig 7B.

#### Low level of excitation: Release-on-escape

**Fig 8:**
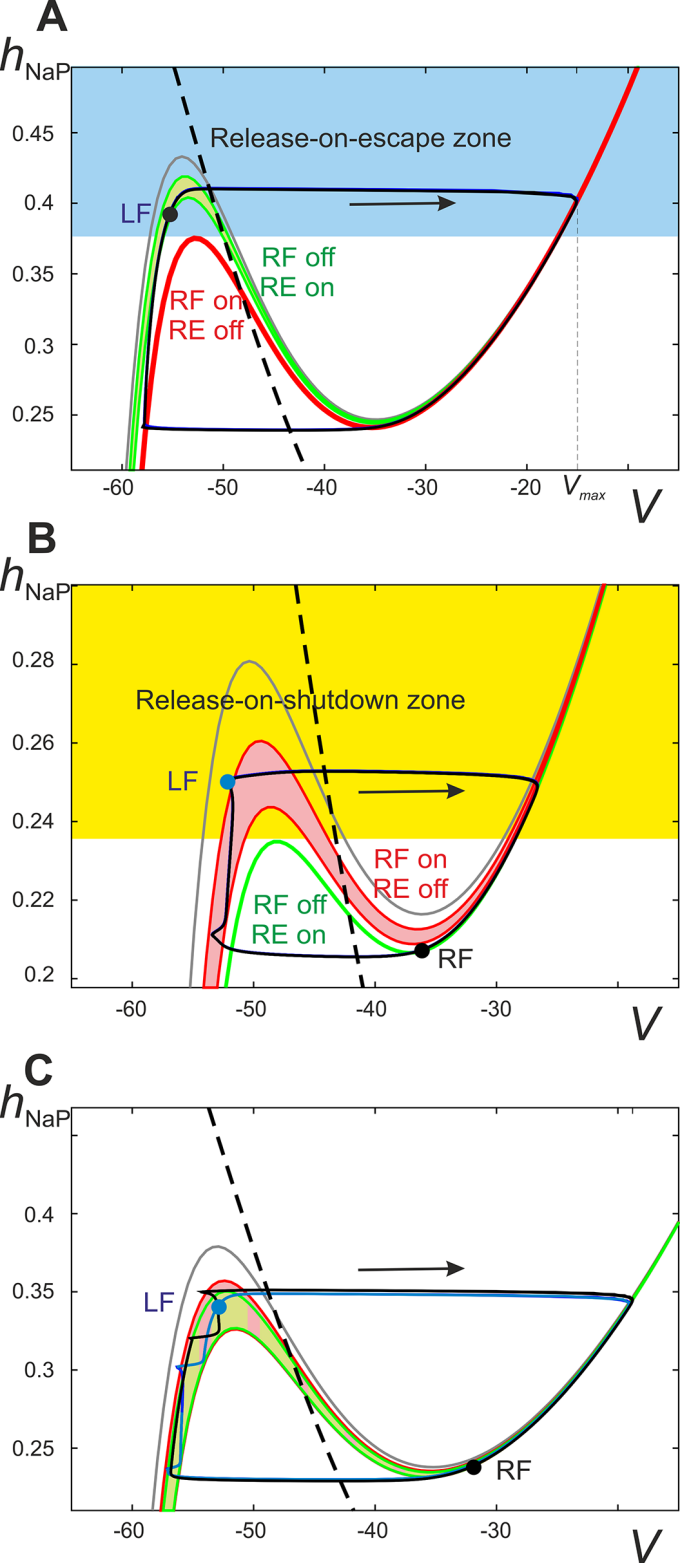
Fast-slow decomposition of the model dynamics when the inhibitory V0_D_ pathways are deleted. Projection of the phase portrait of the model onto the (*V*, *h*_*NaP*_) plane at *α* < X_6_. Sample positions, or image points, of flexor centers are depicted by the black (RF) and blue (LF) circles. **A.** Input from the right extensor center (RE) to the left flexor centers (LF) is represented as a sleeve of nullclines ranging from the maximal RE activity (lowest green nullcline) to minimal RE activity (highest green nullcline). The maximal and minimal RE activities occur at the beginning and end of the RF inactive phase, respectively. The red nullcline represents the maximal excitation to the LF from the RF when the RF is active (labeled as “RF on/RE off”). This maximum occurs immediately after the RF activates and begins to excite the LF via the V3 connection. Hence, for any of the LF's initial conditions above the left knee of the red nullcline (the blue area) the activation of the RF will immediately result in activation of the LF as well (release-on-escape). For low enough values of *α* the left knee of the green band is higher than the left knee of the red nullcline. This makes it possible for the LF to climb high enough during the inactive phase of the RF to find itself in the blue area by the time of escape of the latter. Once the RF activates, the LF activates as well thus stabilizing the regime of synchronous oscillations. **B.** *α* > X_8_ the red and green nullclines interchange their positions so that the left knee of the red band is now higher than the left knee of the green nullcline. Accordingly, it is no longer possible for the LF to get above the left knee of the low red nullcline, while moving along the left branch of the green nullcline, thus making the release-on-escape mechanism impossible and excluding synchronized behavior (see Fig 7B). Instead, such a configuration enables the release-on-shutdown mechanism, because starting at initial conditions above the left knee of the green nullcline (the yellow area) the LF immediately activates upon the deactivation of the RF. Symmetric alternation (Δ*φ* = 0.5) is stabilized by the release-on-shutdown mechanism given that each flexor center’s image point climbs high enough along the left branch of the red band before the contralateral flexor center deactivates. **C.** Overlap of the flexor and extensor center nullcline bands is denoted with a checkered pattern and underlies the bistability observed in the diagram “**Phase dif. LF-RF**” in Fig 7B. This specific scenario corresponds to the area between X_6_ and X_7_ where flexor center synchronization co-exists with alternation in a manner dependent on the system’s initial conditions. Because of the large overlap of the nullcline bands both the escape-on-release and escape-on-shutdown mechanisms are possible (see text for more detailed description).

At low levels of excitation (*α* < X_6_), activities of flexor centers are synchronized signifying that the reciprocal excitation provided by the excitatory V3 pathways is stronger than inputs from the contralateral extensor centers. We found that there are prerequisites for the release-on-escape mechanism, which are further explained using fast-slow decomposition (Fig 8A). The existence of this regime depends on the maximum voltage (*V*_*max*_) achieved by the leading flexor center upon activation.

Indeed, let’s suppose that both flexor centers are inactive and hence travel along the left branch of the corresponding nullcline (see the one labeled “RF off/RE on” in Fig 8A). When activated, the leading flexor center inhibits the ipsilateral extensor center and excites the contralateral (lagging) flexor center. Therefore, the lagging flexor center’s image point has to jump right to the *V*-nullcline defined by excitation from the now active contralateral flexor center (“RF on/RE off” in Fig 8A). To successfully complete a release-on-escape (i.e. to switch to the high potential branch of “RF on/RE off” nullcline signifying its activation), the lagging flexor center must reach an *h*_*NaP*_-coordinate above the left knee of the red *V*-nullcline. This is only possible if the upper green *V*-nullcline is higher than the red one shown in Fig 8A.

As mentioned, the position of the left knee of the red *V*-nullcline is defined by the voltage *V*_max_ of the leading flexor center after its activation. In turn, the maximum voltage of the leading flexor center inversely correlates with the level of excitation (see “Amplitude (F)” in Fig 7B), because increasing *α*, and therefore *E*_*L*_, lowers the left knee of the upper green *V*-nullcline. Therefore, the activity of the leading flexor center reaches a lower *h*_*NaP*_ coordinate prior to escape and hence lower voltage *V*_max_ upon activation (see the escape part of the trajectory labeled by an arrow in Fig 8A). Decreasing the *V*_max_ of an activated flexor center will reduce the reciprocal excitation via the V3 pathway and therefore raise the red *V*-nullcline relative to the green ones. As *α* continues to increase the left knee of the red *V*-nullcline will eventually exist at higher *h*_*NaP*_-values than the upper green one. Specifically, this event occurs as excitation crosses X_8_ in “Phase dif. LF-RF” diagram in Fig 7B. Accordingly (see Fig 8B), the leading flexor center will not rise to sufficiently high values of *h*_*NaP*_ during the extensor phase (i.e. when moving along the green *V*-nulcline) to produce a large enough *V*_max_ to allow for the release-on-escape mechanism of synchronization. When the release-on-escape mechanism fails, the lagging flexor center begins to travel along the low potential branch of the red *V*-nullcline that is defined by the now active leading flexor center. Such a configuration of nullclines allows for a different synchronizing event, namely, the release-on-shutdown, to provide alternations at higher values of excitation (see below).

#### High excitation: Release-on-shutdown

Increasing excitation (*α* > X_8_, see “Phase dif. LF-RF” diagram in Fig 7B) causes the system to switch to an alternating pattern provided by the release-on-shutdown mechanism. This is depicted in Fig 8B. The active flexor center provides decreasing excitation to the inactive flexor center (due to a gradual decrease of flexor activity within the burst), which causes the left knee of the red *V*-nullcline to progressively rise (thus forming an area between the lower and upper red nullclines). When the activity of the active flexor center terminates, the ipsilateral extensor center is released and provides excitation to the inactive flexor center. This causes the inactive flexor center to activate if its *h*_*NaP*_-coordinate reaches values greater than the left knee of the green *V*-nullcline in Fig 8B (representing contralateral extensor center excitation). The scenario described above can only occur when the left knee of the green *V*-nullcline exists at a lower *h*_*NaP*_-coordinate than that of the upper red *V*-nullcline.

Decreasing excitation (i.e. going backward in “Phase dif. LF-RF” diagram in Fig 7B) slows down the motion of the image point of the inactive flexor center along the red *V*-nullcline. This happens because the reduction in excitation raises the *V*-nullcline's left knee closer to the *h*_*NaP*_ steady state and thus reduces *h*_*NaP*_ ‘s rate of change (see Fig 8C). When excitation is decreased below X_7_, the image point of the inactive flexor center will not travel along the red *V*-nullcline to a high enough *h*_*NaP*_-coordinate (above the left knee of the green *V*-nullcline) at the point when the active flexor center shuts down. This indicates a failure of the release-on-shutdown mechanism and results in flexor center alternations that are not perfectly anti-phase, i.e. Δ*φ* ≠ 0.5 (Fig 8C). Following this failure there is a period of time when both flexor centers are inactive and thus the phase difference between them is not equal to 0.5 (when X_6_ < *α* < X_7_ in “Phase dif. LF-RF” diagram in Fig 7B). Alternations of this nature will heretofore be referred to as “coupled” alternations.

#### Intermediate excitation: Bistability

As seen in “Phase dif. LF-RF” diagram in Fig 7B, bistability exists when X_6_ < *α* < X_8_. Specifically, left-right synchronous activity persists as excitation is increased up to X_8_ and alternations persist until excitation is decreased beyond X_6_. This bistable behavior can be explained with Fig 8C. The bistability occurs because the bands of green and red nullclines have a large area of overlap. The boundaries of the red band are defined by the activity of the contralateral flexor center from the time of its activation (lower border) to shutdown (upper border). Similarly, the lower and upper boundaries of the green band are defined by the maximal and minimal activity of an active contralateral extensor center. This overlap allows for both release-on-escape and release-on-shutdown mechanisms, because the release-on-escape mechanism requires the lower red *V*-nullcline to be lower than the upper green nullcline, and the release-on-shutdown mechanism can operate when the lower green nullcline is lower than the upper red nullcline.

#### Overall bifurcation scenario

At low levels of excitation (*α* < X_6_) in “Phase dif. LF-RF” diagram in Fig 7B, the activity of flexor centers is synchronized. An increase in excitation leads to the disappearance of this regime at *α* = X_8_. The system switches to left-right alternating pattern when *α* is increased beyond X_8_. As *α* is reduced backward from high values a hysteresis is observed: the regime of flexor activity alternations persists through decreasing values of excitation until *α* = X_6_ when synchronous flexor oscillations emerge as the only possible pattern of network activity. In summary, after removal of V0_D_ pathways the system demonstrates left-right synchronization at low levels of excitation (*α* < X_6_) and left-right alternations at high levels of excitation (*α* > X_8_). For intermediate values (X_6_ < *α* < X_8_) both behaviors may be reproduced by selecting specific initial conditions.

In summary, the dynamical mechanisms involved in this scenario are the following. At low excitation levels, the amplitude of flexor bursts is relatively high, which makes the V3 excitatory interactions between the flexor centers stronger than the V0_V_-mediated inputs from the contralateral extensor centers (Fig 7). The resulting interaction can be classified as net excitation, thus providing the release-on-escape mechanism (Fig 8A) underlying in-phase synchronization of the flexor centers.

With an increase in *α*, the amplitude of the flexor oscillations reduces thus attenuating the V3-mediated interactions relative to the V0_V_-mediated ones. Eventually this changes the balance between these inputs, so the effective interaction between the flexor centers becomes inhibitory (Fig 8B). Accordingly, at low flexor amplitudes the centers exhibit an alternating oscillatory pattern based on the release-on-shutdown mechanism. This explains why V0_V_ CINs support left-right alternating pattern at high frequencies, but not at low frequencies.

### Summary of Model Performance under Different Conditions

The above analysis of our model is centered on two major regimes characterized by left-right alternation and left-right synchronization (hopping). We have shown that the operating regime depends on the network integrity (presence or lack of particular commissural interactions), level of excitation in the network, and initial conditions. The general conclusion from this analysis is that is in the intact system, the left-right alternation is secured by the V0_D_ CIN pathways at low excitabilities/frequencies and by V0_V_ CIN pathways at high excitation/frequencies. The regimes of operation and patterns exhibited are summarized in Table 2 below:

**Table 2:** Models, operation regimes and patterns.

Model	Level of excitation		
	Low	Intermediate	High
Intact	Alternation	Alternation	Alternation
V0_V_ deleted	Alternation	Bistable	Synchronization/hopping
V0_D_ deleted	Synchronization/hopping	Bistable	Alternation
Both V0 deleted	Synchronization/hopping	Synchronization/hopping	Synchronization/hopping

In the following section we provide a more general description of the network to elucidate the critical elements of the model that lead to the synchronization properties described above.

### Interpretation of the Resuts in Terms of Phase Synchronization

In the model proposed the connections between neurons are relatively weak. In this case, the dynamics of the system may be described in terms of phase synchronization. This approximation implies that the trajectory of each oscillator in its own phase subspace is perturbed negligibly by the interactions between the oscillators, and hence the state of each oscillator can be described by a single variable, phase. Accordingly, taking into account the left-right symmetry of the system, the full set of differential equations describing the system can be reduced as follows in the approximation of asymptotically small connections (see, for example, [59]):

$$\begin{array}{l} {\dot{\phi }}_{1}=\Omega +F(\phi_{1},\phi_{2});\\ {\dot{\phi }}_{2}=\Omega +F(\phi_{2},\phi_{1}), \end{array}$$ (22)

where *ϕ*_1_ and *ϕ*_2_ are the phases of the oscillators, Ω is an oscillation frequency and the function *F(*.,.*)* describes interactions between oscillators. For the phase difference *ϕ* = *ϕ*_2_ − *ϕ*_1_ we will have

$$\dot{\phi }=F(\phi_{2},\phi_{1})-F(\phi_{1},\phi_{2}).$$ (23)

Hypothesizing that the right hand side of this equation depends only on the phase difference, i.e. *F*(*ϕ*_2_, *ϕ*_1_) − *F*(*ϕ*_1_, *ϕ*_2_) = *G*(*ϕ*), allows Eq (23) to be rewritten in a simpler form:

$$\dot{\phi }=G(\phi ).$$ (24)

The synchronized regimes of this system are the fixed (equilibrium) points of this first order differential equation (i.e. solutions of the equation *G*(*ϕ*) = 0) and their stability is defined by the sign of the first derivative *dG*(*ϕ*) / *dϕ* at the equilibrium. Specifically, the point is stable if the first derivative *dG*(*ϕ*) / *dϕ* is negative and the point is unstable if the derivative is positive. If we introduce a potential function as *P*(*ϕ*) = −∫*G*(*ϕ*)*dϕ*, then stable equilibrium points in Eq (24) will be local minima (valleys) of the function *P*(*ϕ*), and unstable points will be local maxima (hills) of *P*(*ϕ*).

Because *G*(*ϕ*) is a 2*π*-periodic function that is odd, i.e. maintaining symmetry about the origin such that –*G*(*φ*) = *G*(−*φ*), its Fourier series expansion may be represented as:

$$G(\phi )=\sum_{k=1}^{\infty }A_{k}\cdot \text{sin}(k\phi ).$$ (25)

Restricting the expansion to the first two terms gives the following equation:

G(ϕ)=A⋅sinϕ−B⋅sin2ϕ=sinϕ⋅(A−2B⋅cosϕ). (26)

This equation always has at least two fixed points at *ϕ* = 0 and *ϕ* = *π* representing synchronization and alternation regimes, respectively. The former is stable whenever *A* – 2*B* < 0, and the latter is stable if *A* + 2*B* > 0. Accordingly, for *A* < −2*B* only the synchronization regime *ϕ* = 0 is stable, for *A* > 2*B* only the alternation regime *ϕ* = *π* is stable, and between these values for −2*B* < *A* < 2*B* both regimes are stable. This inequality has solutions only if *B* > 0, so the second term in the expansion (Eq (26)) with positive coefficient *B* is responsible for the existence of bistability in the system.

Another qualitative conclusion from these speculations is that greater values of the parameter *A* correspond to “less stable” synchronization and “more stable” alternation in a manner referring to these behavior's basins of attraction. Conversely, smaller values of *A* make synchronization “more stable” and alternation “less stable”. It is straightforward to assume that excitatory connections between oscillators contribute to a decrease in *A*, while inhibitory connections increase *A*. In the system under consideration, V3 and V0_D_ commissural pathways mediate the direct excitatory and inhibitory connections between the flexor centers, respectively, and V0_V_ pathways represent excitatory connections from the contralateral extensor to each flexor center, and hence can be considered as effectively inhibitory pathways between the flexor centers. Accordingly, the parameter *A* can be constructed from synaptic weights of these connections as *A* = −*V*_3_ + *V*_0*V*_ +*V*_0*D*_, where *V*_3_, *V*_0*V*_, and *V*_0*D*_ represent the strengths of the corresponding pathways. Assuming that all considered pathways include interneurons whose excitability is affected by NMDA, we can suggest that the strength of these pathways linearly increases with *α*:

$$\begin{array}{l} V_{3}=V_{3}^{0}+k_{3}\cdot \alpha ;\\ V_{0V}=V_{0V}^{0}+k_{0V}\cdot \alpha ;\\ V_{0D}=V_{0D}^{0}+k_{0D}\cdot \alpha . \end{array}$$ (27)

Accordingly, we can rewrite an expression for *A* as:

$$A=-V_{3}^{0}+V_{0V}^{0}+V_{0D}^{0}+\alpha \cdot (-k_{3}+k_{0V}+k_{0D}).$$ (28)

**Fig 9:**
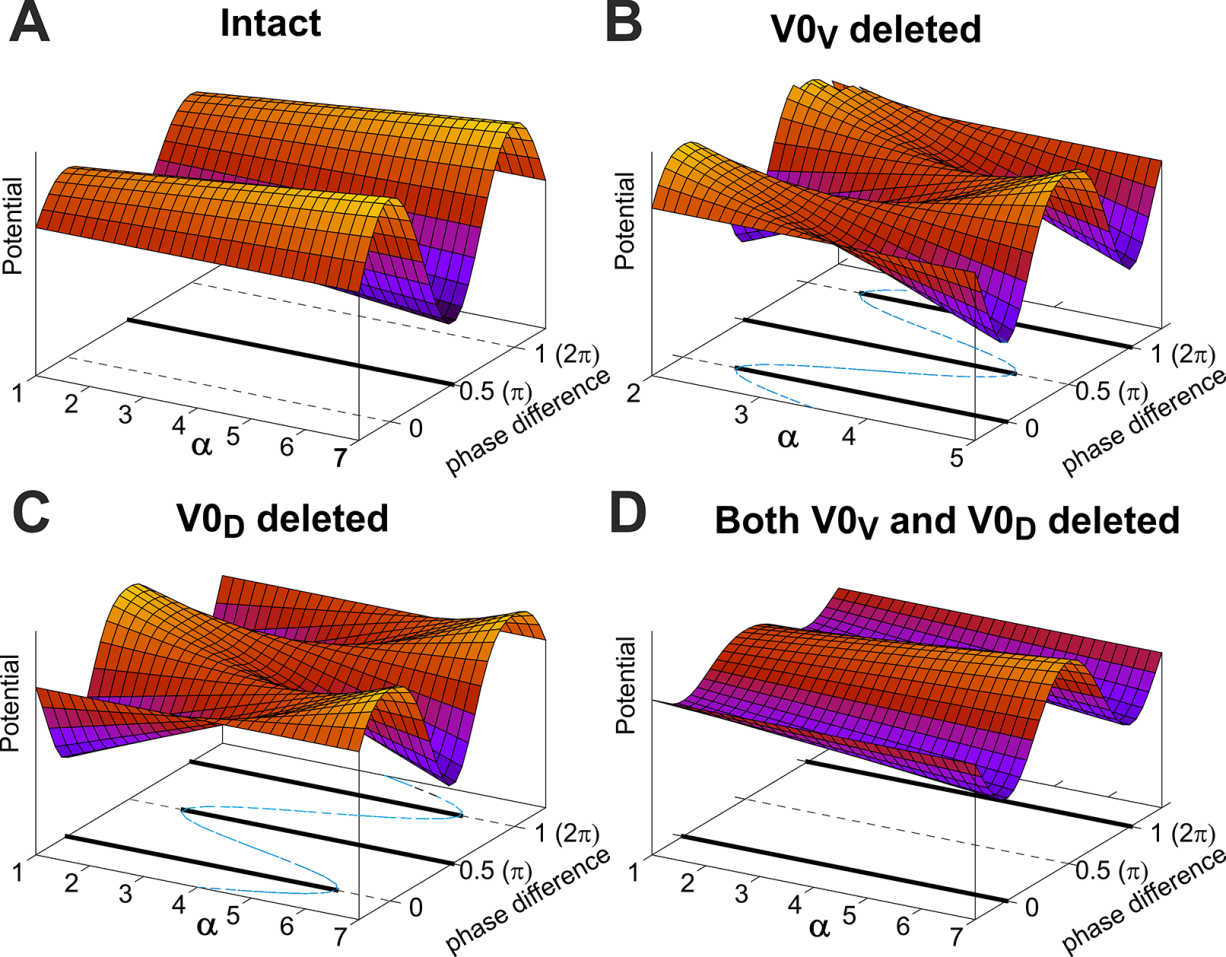
Interpretation of system behavior in terms of phase synchronization. A potential function for Eq (24), *P*(*ϕ*) (see text for the definition), is depicted as a function of phase difference and the excitation parameter, *α*, together with the bifurcation diagram (at the bottom) for each case: **A.** Intact model; **B.** V0_V_ connections are deleted; **C.** V0_D_ connections are deleted; **D.** Both V0 connections are deleted. Note that the phase difference is normalized to 1, i.e. Δ*φ* = *φ* / 2*π* and Δ*φ =* 0.5 of the phase difference corresponds to alternation (*ϕ* = *π*), whereas Δ*φ* = 0 or 1 corresponds to synchronization (*φ* = 0 or 2*π*). Bifurcation diagrams show the coordinates of the fixed points depending on *α* on (*α*, *ϕ*)-plane. Stable equilibriums are shown by thick solid lines, and unstable states are on the dashed lines. Same states can be recognized as valleys and hills on the potential surface at particular values of *α*. For example, in the case of V0_D_ deleted, at *α* = 1 we start with out-of-phase (alternating) regime (phase dif. Δ*φ* = 0.5) being a hill. As *α* increases, the surface bends into a shallow valley around this point, which progressively gets deeper. In contrast, valleys that correspond to in-phase synchronization (phase dif. Δ*φ* = 0, 1) become more and more shallow, and eventually turn to hills, which signifies stability loss. Parameters used to calculate the potential surfaces and bifurcation diagrams: $V_{3}^{0}=4$; $V_{3}^{1}=2$; $V_{0V}^{0}=0$; $V_{0V}^{1}=3$; $V_{0D}^{0}=11$; $V_{0D}^{1}=0$. See text for more details.

In these terms the bifurcation scenarios described above allow for an elegant qualitative interpretation (see Fig 9A–9D). In the case when both subtypes of V0 pathways are deleted $V_{0V}^{0}=V_{0D}^{0}=0$, *k*_0*V*_ = *k*_0*D*_ = 0, and the parameter $A=-V_{3}^{0}-\alpha \cdot k_{3}$ is always negative and becomes even more negative with increasing *α* due to the negative slope. Accordingly, the synchronization regime remains stable for all values of *α* and the alternation regime is always unstable (see Fig 9D).

Let’s now consider the case when only V0_V_ pathways are deleted. In this case $V_{0V}^{0}=0$ and *k*_0*V*_ = 0. Therefore, $A=-V_{3}^{0}+V_{0D}^{0}+\alpha \cdot (-k_{3}+k_{0D})$. If we assume that the connections mediated by inhibitory V0_D_ pathways have a much stronger basal component ($V_{0D}^{0}>V_{3}^{0}$), but weaker dependence on *α* than the excitatory V3 pathways (*k*_0*D*_ < *k*_3_), then the baseline activity of the system (with small *α*) will be the opposite of the case when both V0 pathways are deleted since *A* can now be positive and large enough. With an increase in *α* the *α*-dependent term will eventually prevail and we will get the same situation as when all V0 pathways are deleted. In Fig 9B this scenario can be seen on the bifurcation diagram shown at the bottom on the (*α*, *ϕ*)-plane. We start, when moving from smaller to larger values of *α*, with the synchronization regime unstable and alternation regime stable. Then the fixed points *ϕ* = 0, 2*π* become stable through the supercritical pitchfork bifurcation and new unstable fixed points appear which now separate the attraction basins of synchronization and alternation regimes (shown by dashed blue line). As *α* further increases these points move towards, and eventually merge to, *ϕ* = *π* making it unstable. On the surface described by the potential function, *P*(*ϕ*), this scenario manifests itself by the valley at *ϕ* = *π* (Δ*φ* = 0.5) where the surface becomes progressively more shallow and finally becomes a hill (see *α* close to 5).

With V0_D_ deleted, the expression (28) takes the form: $A=-V_{3}^{0}+V_{V}^{0}+\alpha \cdot (-k_{3}+k_{0V})$. Here we make an opposite assumption, i.e. V0_V_ interactions have a smaller basal component than V3 pathways, i.e. $V_{0V}^{0}<V_{3}^{0}$, but stronger *α*-dependent component, i.e. *k*_0*V*_ > *k*_3_. Accordingly, the baseline in this case coincides with the case when both V0 pathways are deleted, i.e. we see stable synchronous and unstable alternation regimes at low values of *α* (see Fig 9C). As *α* increases the parameter *A* decreases due to its negative slope. This ultimately results in switching to the stable synchronization regime. Qualitatively this scenario is similar to the previous one but reversed (see the bifurcation diagrams on the (*α*, *ϕ*)-plane in Fig 9C).

Finally, in the intact system both the intercept and the slope in Eq (28) are positive. The former is due to the fact that $V_{0D}^{0}>V_{3}^{0}$, and the latter is because *k*_0*V*_ > *k*_3_. Accordingly, *A* is always positive and only the alternating (out-of-phase synchronization) regime when *ϕ* = *π* (Δ*φ* = 0.5) is stable (see Fig 9A).

In summary, this simplified model offers an explanation for why elimination of functionally similar interactions provided by V0_V_ and V0_D_ commissural pathways has such dramatically different effects. Based on our analysis, this may happen if the excitatory interactions between left and right rhythm generators are more dependent on (and change with) general neuronal excitation in the network than the inhibitory interactions.

### Asymmetric Changes in Flexor and Extensor Phase Durations with Locomotor Step Cycle

**Fig 10:**
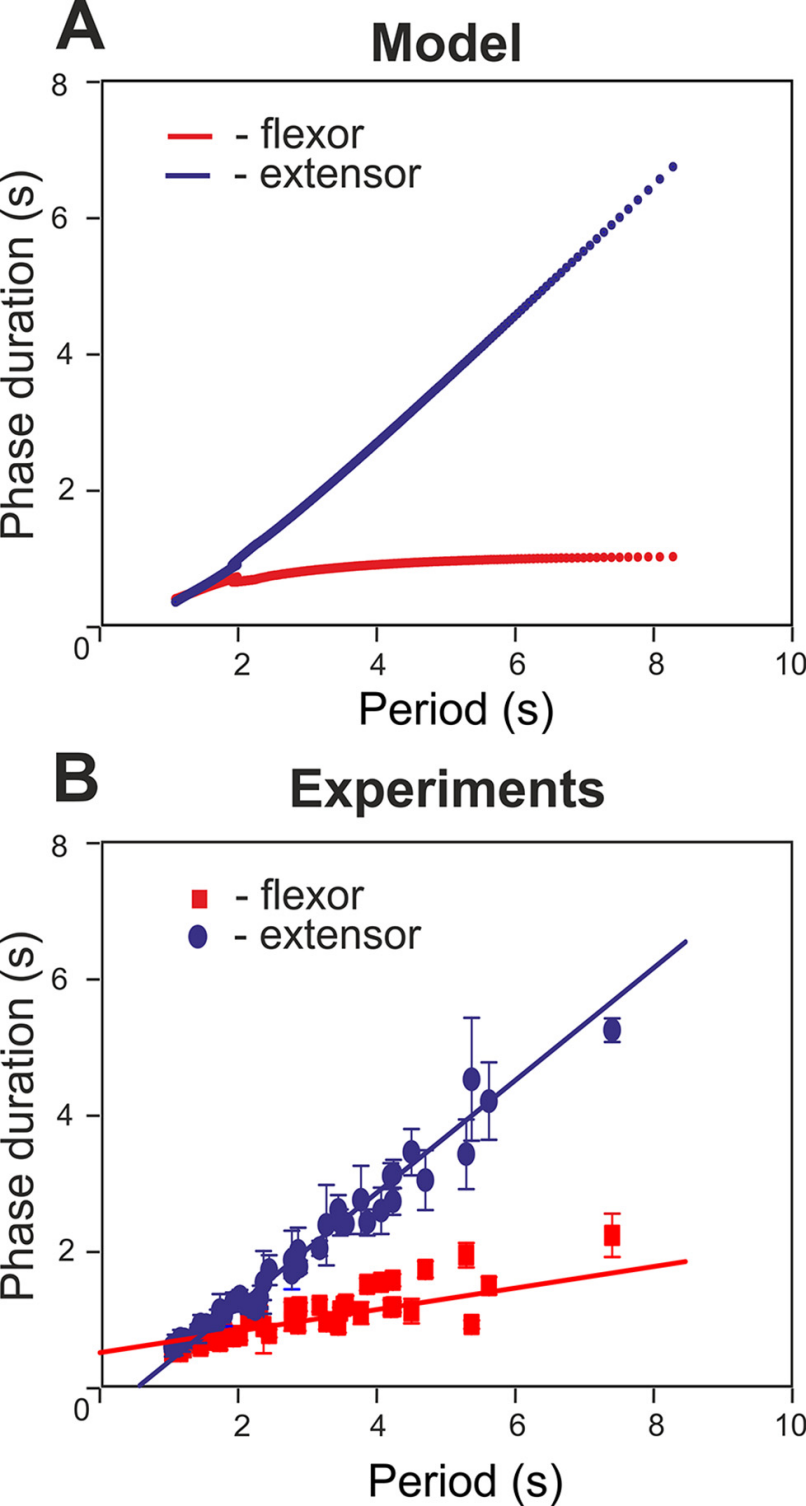
Changing the flexor and extensor phase durations with oscillation period. **A.** Graphs were built for the intact model by varying excitation (*α*). **B.** Flexor and extensor phase durations vs. step cycle period from wild-type mice spinal cord preparations (n = 11). The step cycle period was varied by application of NMDA. Data were obtained from the recording originally published in [37].

One of the most important characteristics of locomotion is the relative change in the flexor and extensor phase durations with changes in the locomotor speed or step cycle period [60,61]. Fig 10A shows how the durations of both phases in our intact model change with an increase in the locomotor period (slowing down locomotor oscillations). These data demonstrate clearly asymmetric changes in phase durations with variations of the step cycle, so that changes in the duration of the flexor phase are significantly less than changes in the duration of the extensor phase. This means that an increase in locomotor frequency mainly occurs due to shortening the extensor phase. Fig 10B shows a similar diagram representing the changes in flexor and extensor phase durations vs. step cycle period built using the recordings from wild-type mouse spinal cord preparations. One can see that the asymmetric changes in phase durations in experimental studies are qualitatively similar to those in our simulations.

### Frequency-Dependent Role of V0_V_ and V0_D_ Commissural Interactions

#### Frequency-dependent role of V0_V_ and V0_D_: Comparison with experimental data

**Fig 11:**
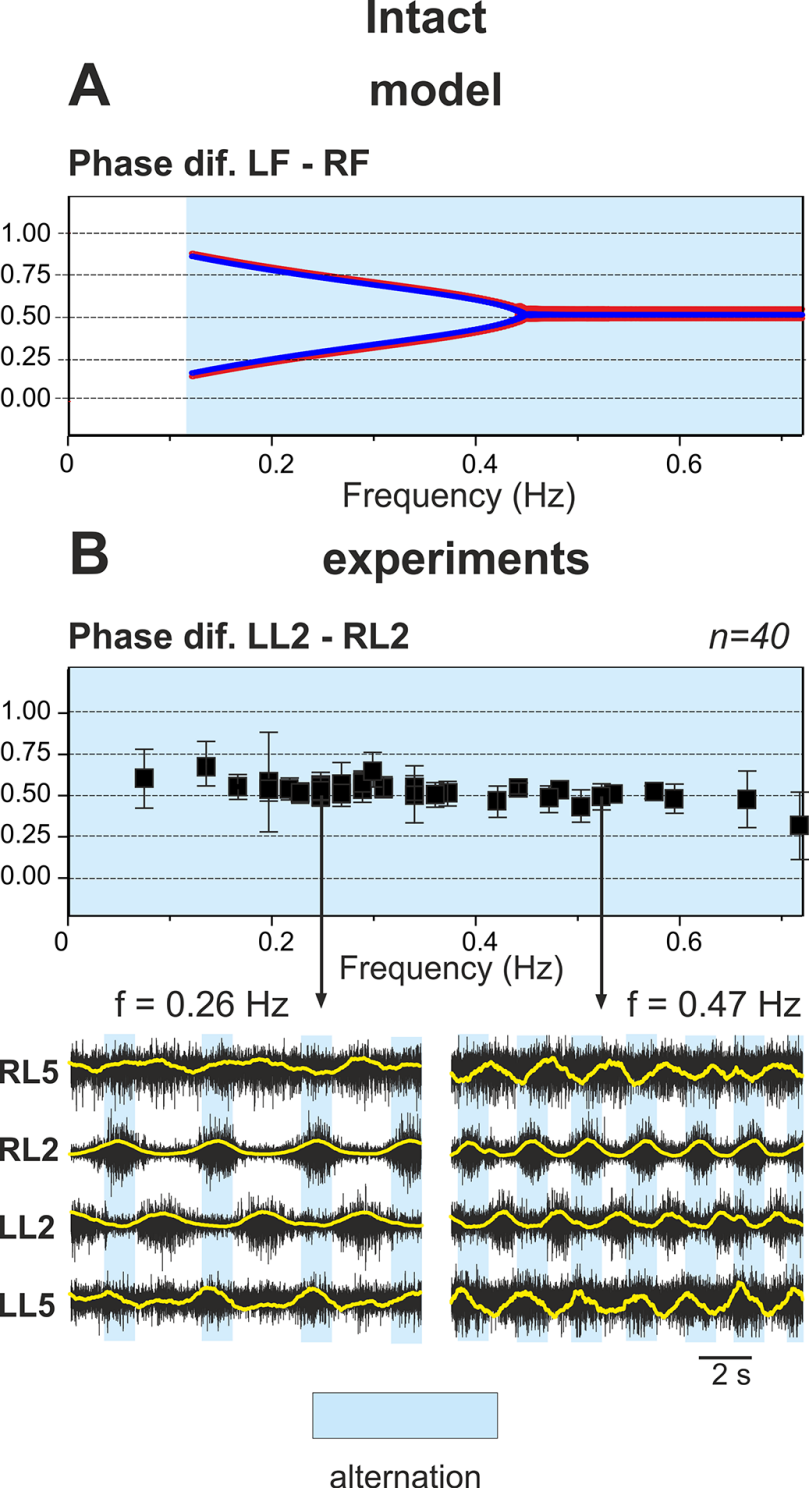
Phase-vs.-frequency bifurcation diagram for the intact model: Comparison with experimental data. **A.** The phase-vs.-frequency bifurcation diagram showing the dependence of phase difference between LF and RF on the oscillation frequency (transformed from “**Phase dif. LF-RF**” diagram in Fig 3B). For frequencies greater that 0.45 Hz the model demonstrated symmetric left-right alternation of flexor activity with Δ*φ* = 0.5. For lower frequencies the flexors exhibited coupled alternation (see legend to Fig 3B and the text for details). **B.** The phase-vs.-frequency diagram summarizing experimental data from spinal cord preparations of wild type mice based on recordings from left (LL2) and right (RL2) lumbar roots. In this diagram, each square represents the mean phase difference between activities recorded from LL2 and RL2 in each preparation (n = 40); error bars in each square indicate the standard deviation. Examples of two experimental recordings from RL5, RL2, LL2 and LL5 roots (raw recordings—black, and rectified—yellow) are shown at the bottom (vertical arrows indicate correspondence to the particular points in the upper diagram). Phase differences in all experiments remain close to 0.5 signifying alternating flexor behavior (details of recordings, measurements and processing can be found in [37]).

**Fig 12:**
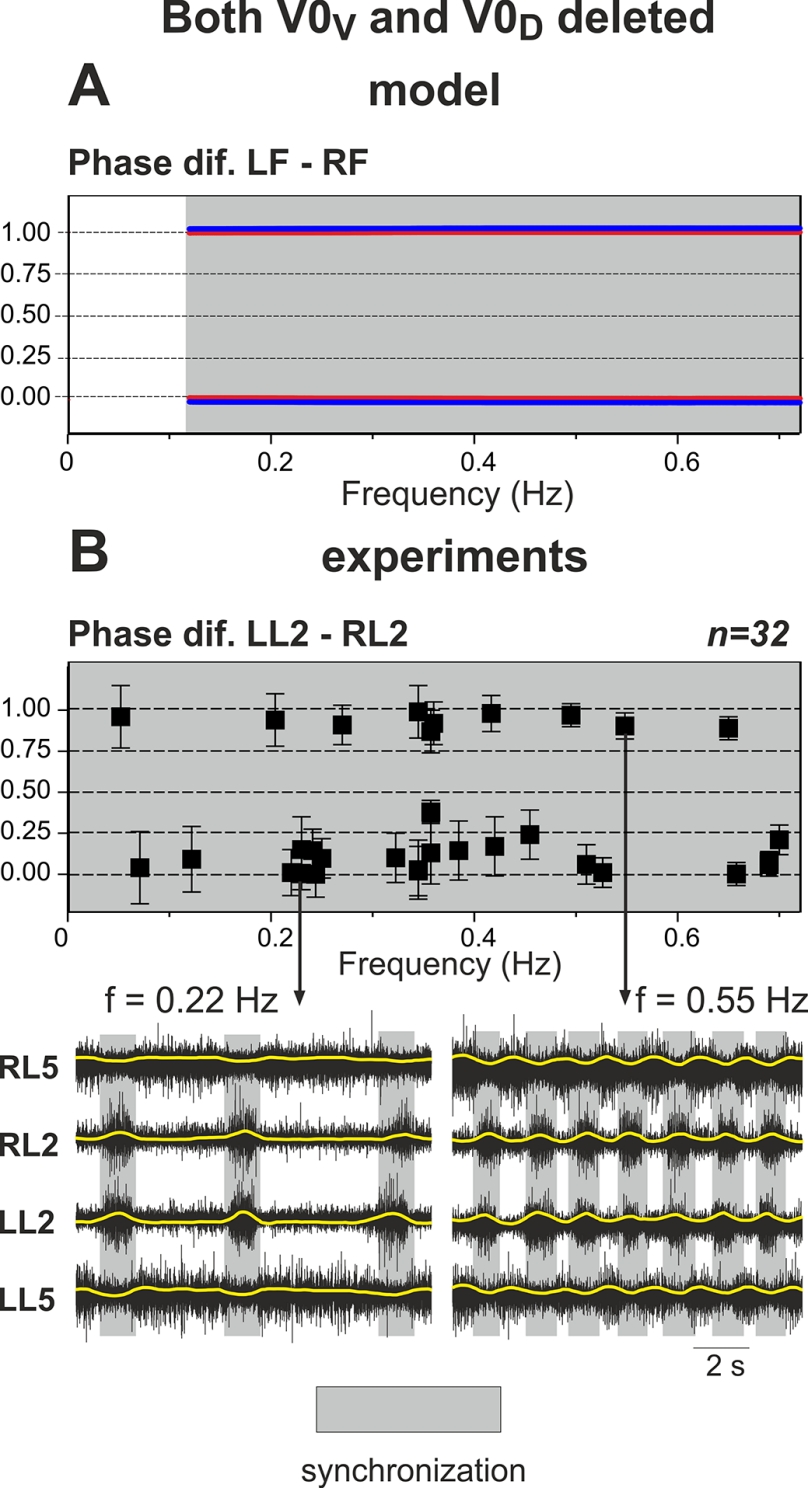
Phase-vs.-frequency bifurcation diagram for the model with both V0 pathways deleted: Comparison with experimental data. **A.** The phase-vs.-frequency bifurcation diagram for the model with both V0 pathways deleted (transformed from “**Phase dif. LF-RF**” diagram in Fig 4B). For all frequencies perfect left-right synchronization (hopping) is observed (Δ*φ* = 0 or 1). **B.** The phase-vs.-frequency diagram summarizing experimental data from spinal cord preparations of knockout mice with deleted V0 commissural interneurons based on recordings from left (LL2) and right (RL2) lumbar roots. As in Fig 11B, each square represents the mean phase difference between LL2 and RL2 activities in each preparation (n = 32). Examples of two experimental recordings from RL5, RL2, LL2 and LL5 roots (raw recordings—black, and rectified—yellow) are shown at the bottom (vertical arrows indicate correspondence to the particular points in the upper diagram). Phase difference remains close to 0 or 1 signifying synchronous flexor behavior. Reproduced from Talpalar et al. [37], Fig 2C and 2D.

**Fig 13:**
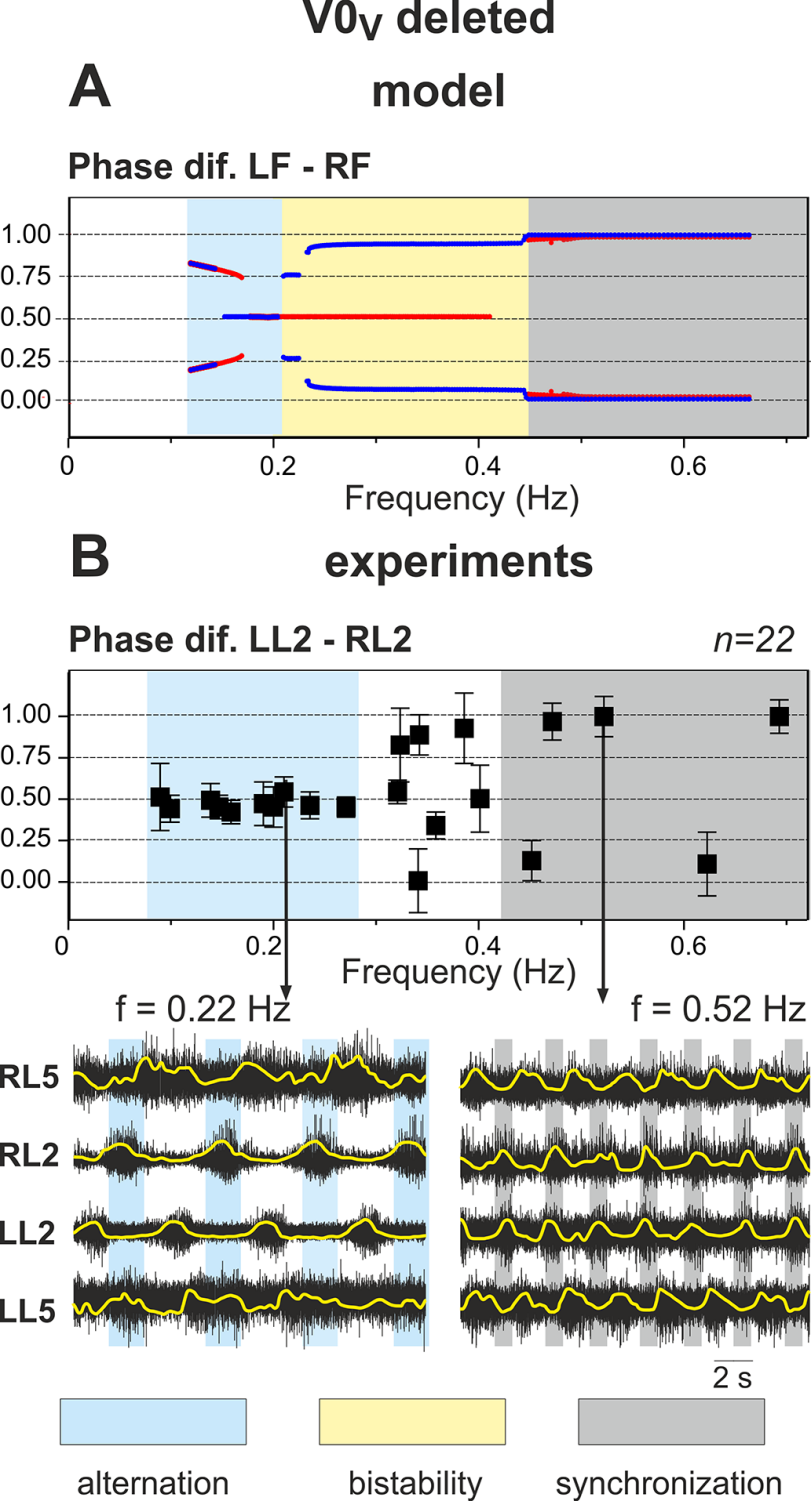
Phase-vs.-frequency bifurcation diagram for the model with V0_V_ pathways deleted: Comparison with experimental data. **A.** The phase-vs.-frequency bifurcation diagram for the model with only V0_V_ pathways deleted (transformed from “**Phase dif. LF-RF**” diagram in Fig 5B). Alternating behavior occurs at low frequencies and hopping at high frequencies with an area of bistability separating the two (between approximately 0.2 and 0.42 Hz). **B.** The phase-vs.-frequency diagram summarizing experimental data from spinal cord preparations of knockout mice with selective removal of V0_V_ commissural interneurons based on recording from left (LL2) and right (RL2) lumbar roots. As in Figs 10B and 11B, each square represents the mean phase difference between LL2 and RL2 activities in each preparation (n = 22). Examples of two experimental recordings from RL5, RL2, LL2 and LL5 roots (raw recordings—black, and rectified—yellow) are shown at the bottom (vertical arrows indicate correspondence to the particular point in the upper diagram). Phase difference is near 0.5 at low frequencies and close to 0 or 1 at high frequencies signifying alternating and synchronous flexor behavior, respectively. For the intermediate frequencies (from about 0.3 to 0.42 Hz) phase differences are widely distributed and intermittently synchronized behavior is observed. Reproduced from Talpalar et al. [37], Fig 4D and 4E.

**Fig 14:**
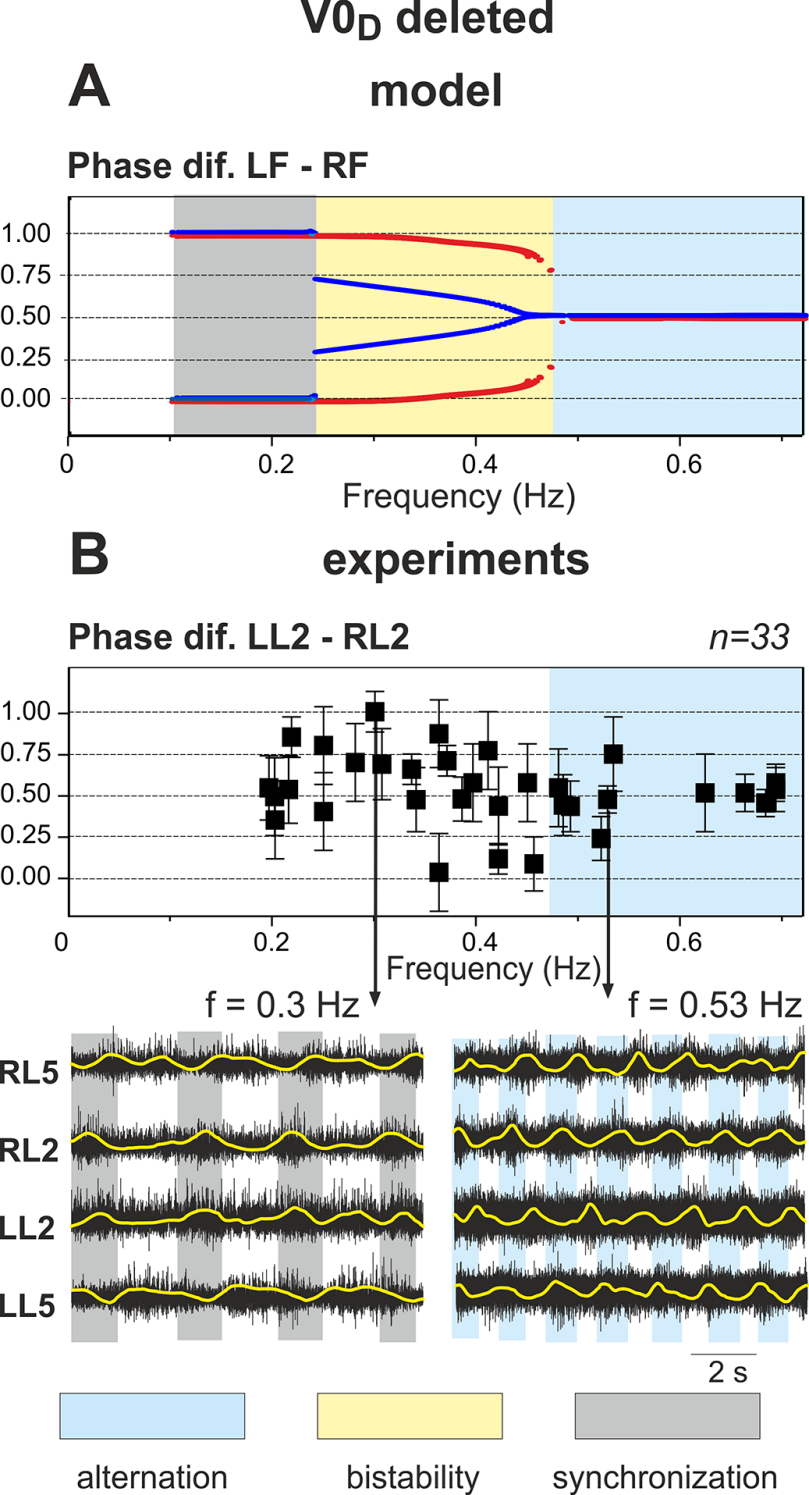
Phase-vs.-frequency bifurcation diagram for the model with V0_D_ pathways deleted: Comparison with experimental data. **A.** The phase-vs.-frequency bifurcation diagram for the model with only V0_D_ pathways deleted (transformed from “**Phase dif. LF-RF**” diagram in Fig 7B). Alternating behavior occurs at high frequencies and hopping at low frequencies with an area of bistability separating the two (between approximately 0.24 and 0.47 Hz). **B.** The phase-vs.-frequency diagram summarizing experimental data from spinal cord preparations of knockout mice with selective removal of V0_D_ commissural interneurons based on recording from left (LL2) and right (RL2) lumbar roots. As in previous diagrams, each square represents the mean phase difference between LL2 and RL2 activities in each preparation (n = 33). Examples of two experimental recordings from RL5, RL2, LL2 and LL5 roots (raw recordings—black, and rectified—yellow) are shown at the bottom (vertical arrows indicate correspondence to the particular points in the upper diagram). Left-right alternating behavior (phase difference about 0.5) is observed at high frequencies (more than 0.47 Hz). At lower frequencies phase differences are widely distributed and intermittently synchronized behavior is observed. Reproduced from Talpalar et al. [37], Fig 3D and 3E.

Recent experimental studies [37] revealed the specific frequency-dependent contribution of V0_V_ and V0_D_ commissural pathways to the left-right alternation of activity in the spinal cord. In Figs 3–5 and 7 we analyzed the relationship of flexor-flexor phase difference to the variable *α* that defined neuronal excitation and conditionally represented changes in NMDA concentration (Figs 3B, 4B, 5B, and 7B). To explicitly compare our simulations with the results of experimental studies, we needed to modify bifurcation diagrams “Phase dif. LF-RF” representing phase difference depending on *α* in Figs 3B, 4B, 5B, and 7B to the diagrams representing the same LF-RF phase difference depending on oscillation frequency. Such transformed phase-vs.-frequency bifurcation diagrams are shown in Figs 11A, 12A, 13A, and 14A, respectively, placed at the top of the diagram representing the corresponding experimental data from the studies of Talpalar et al. [37]. It should be noted that the phase-vs.-frequency bifurcation diagrams, showing LF-RF phase difference vs. frequency, are similar to the original “Phase dif. LF-RF”diagrams, plotting LF-RF phase difference vs. *α*, with only small differences occurring when the frequency as a function of *α* has noticeable non-linearity.

#### Intact model

For the intact case, the phase-vs.-frequency diagram (transformed from the corresponding “Phase dif. LF-RF” diagram in Fig 3B) is shown in Fig 11A. Fig 11B represents similar phase diagrams built from experimental studies on spinal cords from wild-type mice with two examples of root recordings at one low and one high frequency shown at the bottom. Similar to the experimental studies, our model in the intact case (Fig 11A) demonstrated stable flexor-extensor alternation at any locomotor frequency. However, the experimental data roughly depicted a perfect anti-phase (“symmetric”) regime with the left-right phase difference around Δ*φ* = 0.5 at any frequency. This implies that each flexor burst appears approximately in the middle of the contralateral extensor bursts (Fig 11B). In a modeling diagram for the intact case (Fig 11A) the activities of left and right flexors also alternate at all frequencies, i.e. they are never active at the same time. However, this alternation at low frequencies may occur with the phase difference, Δ*φ*, noticeably different from 0.5. Moreover, at these low frequencies, the model demonstrates a bifurcation and two stable regimes, which coexist and depend on which flexor center, left or right, is activated first and inhibits the other one. The phase differences in these cases are approximately equal to the normalized flexor burst duration (up to 0.2) for one branch and 1 minus the normalized flexor burst duration (up to 0.8) for another branch (see Fig 1A). With frequency increase, both branches approach each other and (at approximately 0.45 Hz) merge to a common Δ*φ* = 0.5. The mechanism of these “coupled” alternations with Δ*φ* ≠ 0.5 is based on a post-inhibitory rebound resulting from the inhibitory V0_D_ interactions. Specifically, when a leading flexor center is active, it inhibits the contralateral flexor center and the latter experiences a rise in the conductance of persistent sodium current during the period of inhibition (because of the increasing *h*_*NaP*_, see Eq (12)). This disparity with experimental data is caused by the simplification used to describe each center as a non-spiking activity-dependent neuron model (see Methods).

#### Removal of both V0 pathways

The phase-vs.-frequency diagram for the model version when both inhibitory V0_D_ and excitatory V0_V_ pathways are removed (transformed from the corresponding diagram in Fig 4B) is shown in Fig 12A. The left and right flexors demonstrate activity with a phase difference of either Δ*φ* = 0 or Δ*φ* = 1, both corresponding to synchronization, for all frequency values. This behavior is fully consistent with the results of experimental studies present in the phase-vs.-frequency diagram (Fig 12B) representing data from the spinal cords of mice with genetically removed V0 CINs (both types) exhibiting a left-right synchronization at any frequency (Fig 12B, [37]).

#### Removal of only V0_V_ connections

A phase-vs.-frequency diagram of the model in the case of selective removal of only V0_V_ connections is shown in Fig 13A (transformed from the corresponding diagram in Fig 5B). At low frequencies (less than 0.21 Hz) the model demonstrates a left-right alternating pattern, and at high frequencies (exceeding 0.42 Hz) it shows a stable left-right synchronized or hopping pattern. Fig 13B represents experimental data from spinal cords of mutant mice with selectively ablated V0_V_ neurons [37]. Similar to our modeling data, the spinal cords of these mice exhibit left-right alternating patterns at low frequencies, below 0.25–0.3 Hz, and a left-right synchronized/hopping pattern at higher frequencies (at 0.42 Hz and higher). Similar to the intact case above, there is a disparity between modeling and experimental data at low frequencies, where the model shows a coexistence of 2 or 3 distinct alternating regimes. This discrepancy has the same origin, i.e. a simplification used in description of each center in the model.

Also, as expected from Fig 5B, the phase-vs.-frequency diagram in Fig 13A has a bistable region at medium frequencies, between approximately 0.21 and 0.42 Hz, in which the activity of left and right flexors may be either alternating or synchronized depending on the initial conditions. The experimental diagram (Fig 13B) also shows that at medium frequencies, between approximately 0.3 and 0.42 Hz, the left-right phase differences encompass a wide range of behaviors between alternation and synchronization and display a large standard deviation suggesting intermittent dynamics. Similar behaviors with varying phase differences could be obtained in our simulations by adding certain sources of noise to the center dynamics. Based on our simulations we suggest that, at medium frequencies (0.3–0.42 Hz), the behavior of the spinal cord preparation from mice with selectively removed V0_V_ CINs corresponds to a bistable state of the model with coexistence of both alternating and synchronization modes.

#### Selective removal of V0_D_ connections

Simulations of the cord with selective removal of V0_D_ pathways result in the phase-vs.-frequency diagram shown in Fig 14A (transformed from the corresponding diagram in Fig 7B). At low frequencies (less than 0.24 Hz) the model demonstrates a left-right synchronized/hopping pattern and at high frequencies (more than 0.47 Hz) shows alternating activity. Similar to the previous case, there is a wide bistable region at medium frequencies, between 0.24 and 0.47 Hz, in which the activity of left and right flexors may be either alternating or synchronized depending on the initial conditions. The corresponding diagram in Fig 14B represents experimental data from spinal cords of mutant mice with selectively ablated V0_D_ neurons [37]. The spinal cords of mice lacking V0_D_ CINs exhibit left-right alternating patterns at high frequencies, higher than at 0.47 Hz and a general lack of alternation at intermediate frequencies (between 0.2 and 0.47 Hz). Unfortunately, the mutants with only V0_D_ neurons removed die at birth from impaired breathing. Therefore, data shown in Fig 14B were obtained from isolated spinal cords of embryos, and these cords were unable to generate stable low frequency oscillations [37]. This may be a reason for differences between modeling and experimental data at low frequencies.

Comparison with simulation data (Fig 14A) allows for the suggestion that at intermediate frequencies the spinal cord preparations from mice with selectively removed V0_V_ CINs also operate in an intermittent regime and can demonstrate episodes of left-right alternating or synchronized/hopping behavior.

#### Rhythmic patterns in knockout mice lacking either V0_V_ or V0_D_ CINs at intermediate frequencies

**Fig 15:**
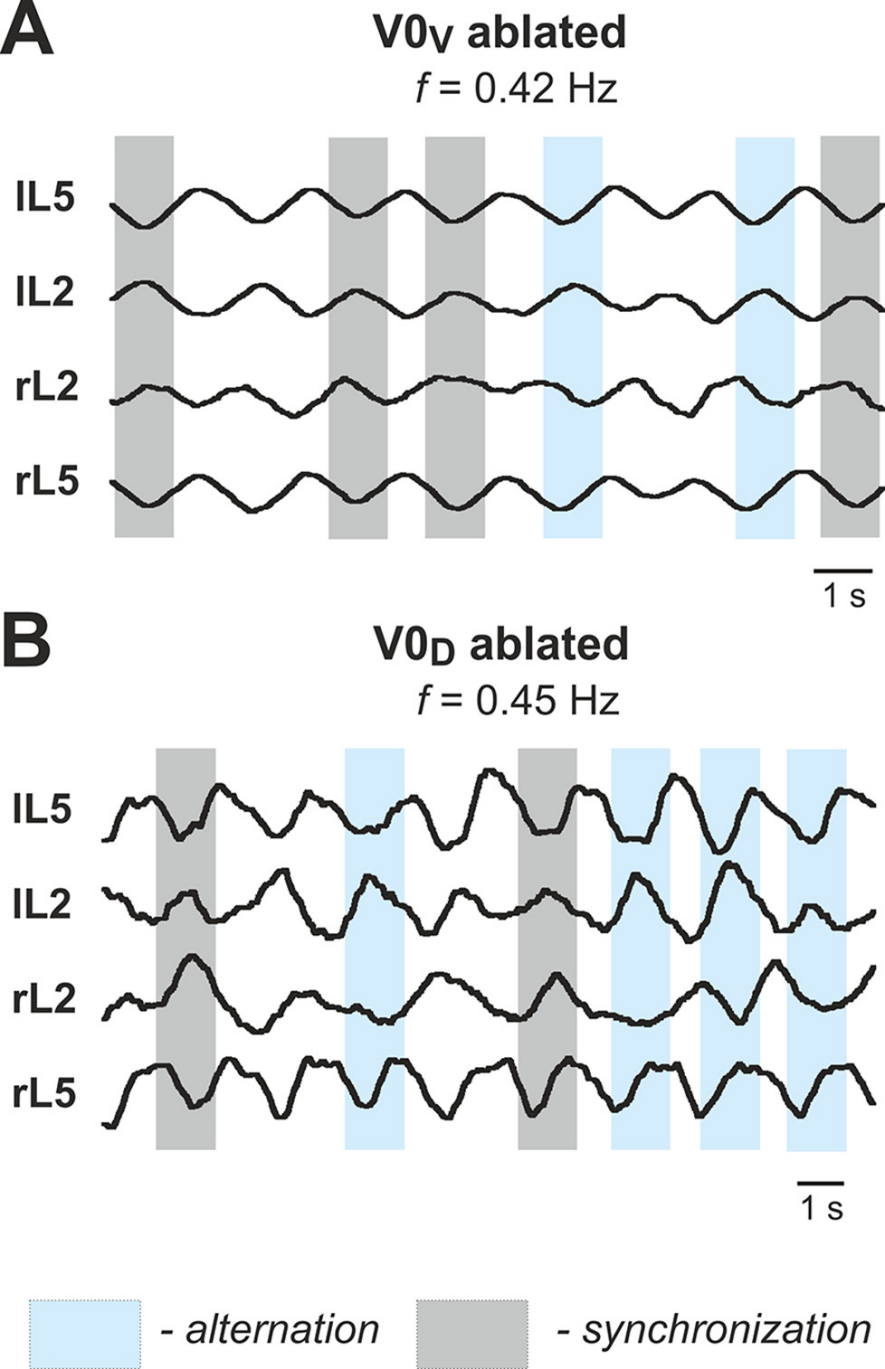
Examples of intermittency in left-right phase relationships in the experimental preparation from mutant mice with selectively ablated V0_V_ and V0_D_ CINs. Recordings from left and right L2 and L5 roots in a V0_V_ (A) and V0_D_ (B) knockout mice at intermediate frequencies demonstrate occasional switching between left-right alternation and synchronization despite maintained values of drug concentration and average frequency.

A prediction generated from the bifurcation diagrams (Figs 5B, 7B, 13A, 14A) is the existence of regions where the system is bistable in cases when only one type of commissural pathway (V0_V_ or V0_D_) is removed. As mentioned previously, in these regions the system can exist in qualitatively different states demonstrating different behaviors, i.e. left-right alternation or synchronization. When locomotor rhythm is generated at an intermediate frequency within the bistable area with a sufficient level of noise, the system can demonstrate occasional switching between left-right alternation and synchronization despite a constant value of drug concentration. Examples of such patterns recorded from experimental studies on knockout mice lacking V0_V_ or V0_D_ CINs are shown in Fig 15, panels A and B, respectively. This provides an additional indirect support for our model design.

## Discussion

### The Use of Theoretical Analysis of Simplified Models for Understanding Left-Right Coordination of Activity in the Spinal Cord

The main objective of this theoretical study was to formulate and analyze a simplified model of the spinal circuitry in order to reproduce and explain recent experimental data concerning frequency-dependent differential contributions of two distinct commissural pathways to the left-right alternation of neural activity in the isolated spinal cord [37]. We used a simplified connectivity scheme adapted from both the experimental data [37] and the previous large-scale computer models [41]. Bifurcation diagrams were constructed for each experimental case to observe the system’s behavior over a large range of excitability (Figs 3–5 and 7). These diagrams allowed us to determine the values of parameters leading to the observed behaviors and provide a strong mathematical explanation of the critical changes seen in left-right phase relationships leading to a normal alternating pattern and to switching to synchronous (hopping) behavior. Moreover, the bifurcation diagrams revealed regions of bistability, where two stable states co-existed for a given set of parameters. To complete our understanding of the dynamics underpinning the transitions, we used fast-slow decomposition methods and generated phase plane diagrams that corresponded to removal of V0_V_ or V0_D_ commissural pathways (Figs 6 and 8). Our analysis showed that left-right alternation and synchronization occurred in both cases when one of the pathways (V0 or V0_D_) was removed, because of the release-on-shutdown and release-on-escape mechanisms, respectively. These findings allowed the interpretation of experimental results in terms of qualitative theory of dynamical systems. In addition, at the intermediate locomotor frequencies, our model exhibited bistable behaviors and predicted a coexistence of both alternating and synchronized regimes, leading to an intermittent alternating/synchronous activity observed in experimental studies (Fig 15).

### Locomotor Pattern Generation and Flexor-Extensor Asymmetry

The exact intrinsic cellular mechanisms involved in rhythmic bursting in the spinal cord remain unknown. Previous modeling studies suggested that these mechanisms may involve the persistent (or slowly inactivating) sodium current, *I*_*NaP*_, [9,41–46,51,52] and this suggestion has been supported by several experimental studies [52–56]. Although we used the *I*_*NaP*_-dependent mechanism in this model, the results of this study concerning network behavior can be considered independent of the exact cellular mechanism employed. At the same time, it should be noted that some particular features of the model fit to known experimental data specifically due to the *I*_*NaP*_—dependent mechanism incorporated (although they can be provided by other cellular/network mechanisms). These features include (a) a monotonic increase of burst frequency and (b) monotonic decrease of (flexor) burst amplitude with an increase of neuronal excitation (by increasing NMDA or glutamate concentration, see [37,40] and Figs 3–5 and 7), as well as (c) small changes in the flexor phase duration relative to the extensor phase duration at low frequencies (the same figures). These observations provide additional indirect support for the idea that *I*_*NaP*_—dependent mechanisms are involved in the generation of bursting activity in the spinal cord.

A recent study using optogenetics [62] has demonstrated that locomotor-like rhythmic bursting can be induced independently in flexor and extensor networks. This means that both flexor and extensor populations can potentially, under certain conditions, generate endogenous locomotor-like, oscillatory activity. However these findings do not necessary imply that both these populations are in the bursting mode under the conditions of isolated spinal cord preparation with locomotor oscillations induced by drugs or other methods. Moreover, analysis of non-resetting deletions (missing bursts) in this preparation showed that missing flexor bursts were always accompanied by a sustained (tonic) extensor activity, whereas missing extensor bursts occurred without an effect on flexor bursting [45]. Finally, the duration of extensor activity between flexor bursts at low burst frequency can be pretty long, exceeding 5–6 seconds when flexor burst duration is usually less than 2.5 seconds [37], which also implies that intrinsic bursting is unlikely to occur in rodent extensors.

Based on the observations described above, we suggest that each flexor or extensor center can potentially, in certain conditions, intrinsically generate bursting activity. However, we think that under the conditions considered here (i.e., the isolated spinal cord with 5-HT and NMDA-induced locomotor-like oscillations) only the flexor centers (left and right) operate in a bursting mode whereas the extensor centers (left and right) operate in a state of tonic activity (if isolated) and exhibit rhythmic bursting because of rhythmic inhibitory inputs from the corresponding ipsilateral flexor center (see the section “Rhythm-generating Centers and Connections”). This may occur because the mixture of 5-HT and NMDA used to evoke locomotor activity in the isolated spinal cord, excites the extensor centers much stronger than the flexor centers (and hence *E*_*LFO*_ < *E*_*LEO*_, see Fig 2B).

The above suggestion leads to the concept that each (left and right) CPG is asymmetric, so that the flexor center on each side differs from the extensor center not only by a level of excitation described above, but also by the organization of left-right and flexor-extensor interactions (see Fig 1B and weights of interactions in Table 1). As a result of this flexor-extensor asymmetry, the burst frequency and the flexor phase duration are mostly defined by the flexor centers, and hence at low frequencies the duration of the flexor phase may be significantly less than the duration of the extensor phase. Fig 10A shows that when frequency goes down (with decreasing *α*) the increase of step cycle period mainly results from an increase of the extensor phase duration with a very small increase of the flexor phase duration. Interestingly, this phase durations vs. period plot built using simulation data fits well to the plot built using data from experimental studies [37] (Fig 10B). This provides additional support for the idea of an asymmetric CPG employed in our study.

The concept of an asymmetric CPG organization with a dominant role of flexor centers in rhythm generation has been previously suggested by Duysens and Pearson [63–66] and implemented in recent computational models [41,45]. Such asymmetric flexor-extensor phase relationships are consistent with the previous experimental studies in cats [60] and support the idea that this flexor-extensor asymmetry is an inherent property of the CPG [61] rather than a result of afferent influences on a quasi-symmetric CPG [67].

### Frequency-Dependent Contribution of V0_D_ and V0_V_ Commissural Pathways to Support Left-Right Alternation of Neural Activity in the Spinal Cord

Our simulations support the earlier suggestion that left-right alternation is provided by two commissural pathways involving the inhibitory V0_D_ and excitatory V0_V_ CINs [37]. Similar to the experimental [37] and previous modelling [41] studies, our simulations demonstrate that the contribution to left-right alternation of the V0_D_ pathway is dominant at low frequencies and reduces as locomotor frequency increases, whereas the contribution of the excitatory V0_V_ pathway is weak at low frequencies but becomes stronger as frequency increases. The analysis of our models suggests that the different frequency-dependent roles of these pathways are based on the following: (1) a relatively weak dependence of V0_D_ CIN pathways on the neuronal excitability (neuroactive drug concentration), which leads to a net reduction of V0_D_ activity due to the reduction of amplitude of rhythm-generating activity when the oscillation frequency increases; (2) a strong dependence of V0_V_ CIN pathways on the neuronal excitability, leading to the increase of their activity with oscillation frequency. This may be based on the existing data that inputs to V0_V_ CINs are often mediated by ipsilaterally-projecting excitatory V2a neurons [37,68,69] These were shown to increase their activity and recruitment with an increase in neuroactive drug concentration [58,70] and their selective ablation mimics the V0_V_ ablation [70].

### Left-Right Coordination and Control of Locomotor Gait

Our study support the concept that the resultant locomotor pattern and locomotor gait depend on the balance between different commissural interactions, which, in turn, depends on the level of neuronal excitation and locomotor speed dictated by the locomotor task. This conclusion is consistent with a general concept that the expression of a particular left-right coordinated pattern, such as the left-right alternation of activity typical for regular walking or the left-right synchronized activity characteristic for hopping or galloping, depends on the balance between the commissural pathways working for or against synchronization of left and right spinal CPGs. Therefore, under normal conditions this pattern and the corresponding gait can be changed by additional speed-dependent or speed-independent descending or afferent signals to different commissural interneuron populations involved in these pathways.
